# Prior-informed machine learning for clinically interpretable medical diagnosis

**DOI:** 10.3389/fbioe.2026.1973664

**Published:** 2026-10-28

**Authors:** Zhicheng Lin, Dawn C. Walker, Craig Murdoch, Helen E. Colley, Dilly O. Anumba, Zi-Qiang Lang

**Affiliations:** 1 School of Computer Engineering, School of Artificial Intelligence, Jiangsu Second Normal University, Nanjing, China; 2 School of Computer Science, University of Sheffield, Sheffield, United Kingdom; 3 Insigneo, University of Sheffield, Sheffield, United Kingdom; 4 School of Clinical Dentistry, University of Sheffield, Sheffield, United Kingdom; 5 School of Medicine and Population Health, University of Sheffield, Sheffield, United Kingdom; 6 School of Electrical and Electronic Engineering, University of Sheffield, Sheffield, United Kingdom

**Keywords:** Bayesian inference, clinical decision support, interpretable artificial intelligence, medical diagnosis, prior-informed machine learning

## Abstract

Traditional machine learning (ML) in medical diagnostics often suffers from limited clinical interpretability and poor generalization, owing to its black-box, data-driven nature. Here, we present Prior-Informed Machine Learning (PIML), a framework that integrates clinically grounded generative reasoning with data-driven, ML-enabled discriminative modeling through Bayesian inference to provide clinically interpretable, patient-specific diagnostic outcomes. By embedding the optimization of sensitivity and specificity in data-driven diagnosis—requirements for subsequent Bayesian inference—directly into the ML model development process, PIML enables a synergistic integration of machine learning and clinical expertise that cannot be achieved through either data-driven or clinically grounded approaches alone. PIML was validated across three clinical domains—cervical neoplasia detection, high-risk oral lesion detection, and preterm birth detection—where Bayesian inference was systematically employed to integrate electrical impedance spectroscopy (EIS)-based machine learning with domain-specific clinical expertise, thereby enabling more comprehensive and enhanced diagnostic decision-making. Our results demonstrate that PIML generally improves diagnostic performance compared with standard ML approaches and provides competitive or superior performance relative to knowledge-based and feature-level prior integration strategies. This work presents an innovative, systematic framework with strong potential for widespread adoption in addressing medical diagnostic challenges across heterogeneous clinical environments.

## 1 Introduction

Artificial intelligence (AI) technologies, particularly machine learning (ML) and deep learning (DL), have demonstrated strong performance in domains such as medical diagnostics (Tu et al., 2025) and industrial systems (Leite et al., 2024), demonstrating impressive performance in real-world applications. However, the ML model makes decisions based on what is learned from the data. Therefore, if the scenario cannot be covered by the data used for the model training, then the decision-making may not be accurate. When ML models are applied to classification problems, the accuracy-related performance is often evaluated using sensitivity and specificity, which are widely adopted by commercially available medical diagnostic products (Nishtar et al., 2021; Sirocchi et al., 2024).

Geoffrey Hinton, often referred to as the “godfather of AI,” noted that the essence of AI lies in pattern learning rather than logical reasoning (Hinton, 2024) and also that ‘learning, not knowledge, was the engine of intelligence (Rothman, 2026). Neural networks (NN) extract abstract features from data rather than perform inference. Once weights are learned, they produce results by applying the NN model to process input data, not through step-by-step reasoning and analysis. This fundamental characteristic of current AI systems explains their strong interpolation abilities but poor extrapolation performance when confronted with unfamiliar data (Bonnasse-Gahot, 2022; Wang et al., 2023). Numerous studies have demonstrated a significant decline in predictive accuracy when ML models are tested on out-of-distribution samples, such as data from different hospitals or populations (Duta et al., 2025; Yang et al., 2022; Zech et al., 2018). Recently Orouji et al. also highlighted that biological ML models trained using data from one cohort or laboratory frequently fail to generalize to other datasets because of both technical and biological distribution shifts (Orouji et al., 2024). In medical imaging, Yang et al. demonstrated across five globally sourced chest X-ray datasets that expert-level vision–language models systematically underdiagnosed patients from historically marginalized demographic groups compared with board-certified radiologists (Yang et al., 2025). These findings underscore the limited generalizability of current AI systems across heterogeneous clinical environments and highlight the fundamental difficulty of maintaining stable diagnostic performance when deployment conditions differ from those represented during model development.

It is worth noting that in AI research, learning and reasoning are often pursued as distinct research directions—namely, machine learning (ML) and knowledge representation and reasoning (KRR) (Baaj et al., 2024). The two domains have traditionally evolved independently. Although recent studies have explored the integration of medical prior knowledge, such as clinical guidelines, statistical rules, and physician expertise into ML model training (Sirocchi et al., 2024; Fang et al., 2024; Jiang et al., 2020; Choi et al., 2016; Leiser et al., 2023; von Rueden et al., 2021; Stewart and Ermon, 2017), most practical implementations still rely on standard model training, in which prior knowledge and data are combined first and then fed into the training stage. Representative examples include CancerVar, which integrates clinical guidelines with Deep Learning (DL) for the clinical interpretation of somatic cancer variants (Li et al., 2022), and PathTIGR, which incorporates curated biological pathway topology into a graph-based DL framework for interpretable prediction of immunotherapy response (Li et al., 2026). Some studies introduce knowledge-based penalty terms into the loss function (Sirocchi et al., 2024; von Rueden et al., 2021). These terms quantify inconsistencies between the model’s predictions and known domain rules, effectively guiding optimization toward clinically consistent outputs. Stewart and Ermon proposed a label-free supervision framework in which domain knowledge, such as physics equations or causal dependencies, is encoded directly into the loss function (Stewart and Ermon, 2017). This allows the model to learn from unlabelled data by minimizing discrepancies between its outputs and known structural priors. While powerful, these methods are typically task-specific, rely on predefined constraint formulations, and may not generalize well across clinical domains with complex, implicit forms of prior knowledge. This creates a need for a more flexible mechanism through which clinical knowledge can influence diagnostic decisions without being explicitly encoded into model architecture or training objectives.

From a broader perspective, most existing approaches remain fundamentally data-driven and are ultimately constrained by the availability, quality, and representativeness of the underlying data. They essentially aim to optimize model weights during backpropagation when, for example, training a NN model. Once the ML model has been trained and parameters are fixed, these weights remain unchanged. As a result, when the model is applied to different scenarios or cohorts, the same learned parameters are still used, regardless of potential shifts in data distribution or clinical context. This can lead to poor model performance and reduced generalizability, particularly in real-world medical settings where patient populations, diagnostic practices, and disease profiles may vary significantly over time and across countries and regions.

Another major limitation of current ML models is their ‘black-box’ nature and lack of interpretability and explainability, which undermines trust and hinders their adoption in clinical applications (Shen et al., 2019; Liu et al., 2019; Baker et al., 2020; Juravle et al., 2020). Studies have pointed out that although AI is often comparable to physicians in diagnostic accuracy, people still lack trust in it, especially in scenarios dealing with high-risk diseases (Juravle et al., 2020). In addition, most of the existing studies are retrospective, i.e., training and testing are based on existing annotated data, with a lack of prospective systematic verification in the real-world environment (Topol, 2019; Kelly et al., 2019).

Physicians play a central role in diagnosis, continuously refining their knowledge through years of experience, peer collaboration, and ongoing learning through extensive clinical practice, peer consultations, and continuous learning which is often abstract, individual-specific, and difficult to formalize (Ananda Rao et al., 2023; Quinn et al., 2021; Sheikhtaheri et al., 2014; Kunhimangalam et al., 2014). In contrast, traditional expert systems have evolved slowly, partly due to their reliance on static rule settings that quickly become obsolete in the rapidly advancing field of medicine (Hughes et al., 1990). Moreover, these systems face the well-known ‘knowledge acquisition bottleneck’- the difficulty of extracting and systematizing implicit knowledge from clinical experts (Sheikhtaheri et al., 2014; Hughes et al., 1990).

For high-dimensional information and features, such as medical/histological images or complex non-linear digital signals, clinicians are inherently limited in learning abstract patterns existing in data. This is easier for AI models, where ML/DL models have matured into mainstream methodologies, replacing manual work in certain well-defined tasks; work that has enhanced outputs in most engineering and healthcare cases. However, in scenarios that require patient-specific and subjective experience-driven clinical judgement, ML/DL models still fall short of fully substituting human expertise due to limitations caused by data-driven characterization. Consequently, neither single AI models nor traditional expert clinician-led systems are sufficient to meet the complex demands of modern healthcare (Topol, 2019; Hughes et al., 1990). Recent studies suggest that collaborative methods that combine AI models with clinical expertise could improve diagnostic outcomes (Ahuja, 2019; Wa et al., 2016; Lakhani and Sundaram, 2017; Göndöcs et al., 2024; Jospin et al., 2022). These studies suggest that a widespread adoption of AI depends on the degree to which ML can help clinicians make a better and more informed diagnostic decision. However, the challenge is that the accumulated knowledge in the human brain is difficult to formulize and to integrate into AI models. Medical diagnosis is inherently probabilistic, integrating symptoms, signs, laboratory findings, imaging evidence, patient history, and prior medical knowledge to infer the likelihood of disease. In order to fundamentally address this challenge, we propose to apply the well-known Bayes Theorem to combine clinical diagnostic decision-making processes with ML to achieve prior-informed machine learning (PIML) to enhance clinically interpretable medical diagnosis. Here, clinical interpretability refers to the transparency and traceability of the diagnostic updating process, rather than implying that the resulting probabilities have been empirically demonstrated to be perfectly calibrated.

Bayes’ Theorem has been widely integrated with ML approaches in medical applications. For example, BNNs (Jospin et al., 2022) represent a prominent integration of Bayesian inference with neural networks (NNs). However, this study does not directly employ Bayesian inference for classification purposes. Unlike conventional NNs, which optimize fixed point estimates of model parameters, Bayesian neural networks (BNNs) incorporate prior distributions and infer posterior distributions of the network weights during training. Consequently, BNNs learn probability distributions over parameters rather than single-point estimates, enabling uncertainty quantification in predictions. In another study, Bayes’ theorem was applied independently to estimate posterior probabilities using disease prevalence for disease diagnosis (HIV) (Muehlemann et al., 2023). More recently, Chatzimichail and Hatjimihail demonstrated the practical utility of the Bayesian diagnostic approach and provided a software implementation for applying this in medical diagnosis (Chatzimichail and Hatjimihail, 2024). These two studies rely on pre-determined sensitivity and specificity values derived from evidence-based classification performance to conduct Bayesian inference. In parallel, Bayesian-network-based approaches have been developed to incorporate structured clinical expertise and heterogeneous medical information into probabilistic diagnostic models. For instance, Kyrimi et al. proposed medical idioms for clinical Bayesian network development, using reusable expert reasoning patterns to support the construction of clinical Bayesian networks (Kyrimi et al., 2020). Donnat et al. developed a Bayesian hierarchical network for combining multiple heterogeneous and uncertain data sources in medical diagnosis (Donnat et al., 2026). To the best of our knowledge, no previous studies have systematically optimized the sensitivity and specificity of evidence-based classifiers and subsequently integrated these optimized diagnostic characteristics with available prior knowledge to achieve systematically optimized Bayesian inference outcomes.

This study focuses on leveraging Bayesian inference to systematically integrate ML-based diagnoses with clinical diagnostic reasoning. The proposed methodology, termed Prior-Informed Machine Learning (PIML), applies Bayes’ Theorem to combine prior domain knowledge, derived from patient information and clinical expertise, with data-driven ML predictions. In PIML, the ML prediction is not treated as an isolated black-box decision, but as diagnostic evidence that updates a patient-specific clinical prior into a posterior probability of disease. In contrast to ML-based diagnoses, which are typically generated by models trained on limited datasets, clinical reasoning draws on population-level statistics, diverse medical tests, and accumulated disease-specific clinical knowledge and experience. By unifying these complementary sources of information, the proposed approach aims, in principle, to achieve an updated diagnostic outcome that integrates prior clinical information with ML-derived evidence not attainable by either approach alone. To validate the performance of the proposed approach, we applied it to diagnostic challenges across three distinct medical domains: cervical neoplasia detection (Li et al., 2021), high-risk oral lesion detection (Murdoch et al., 2014), and preterm birth detection (Tian et al., 2023), (Narice et al., 2024). These fields were selected to capture a number of examples of clinical knowledge that can be integrated with data-driven ML to support comprehensive medical diagnosis. For cervical neoplasia detection, colposcopy findings and smear test results are incorporated to reflect physician diagnostic assessments, a task that current AI techniques still struggle to replace. For preterm birth prediction, individual prior risk is derived from biological markers and clinical history, particularly prior preterm deliveries. For high-risk oral lesion detection diagnostics, behavioral risk factors such as tobacco and alcohol use play a crucial role in risk assessment (Khan et al., 2020) and provide important epidemiological priors for model interpretation. Such knowledge-based medical diagnoses rely on population-level statistics and the extensive experience of clinicians and therefore cannot be fully replaced by AI models trained on limited datasets. On the other hand, the data used to train the ML models are derived from electrical impedance spectroscopy (EIS) measurements. EIS characterizes the electrical properties of biological tissues and detect impedance changes associated with alterations in the cellular architecture of the cervical and oral mucosal epithelium, thereby facilitating the identification of potentially malignant and malignant lesions in the cervix and oral cavity, respectively. The EIS data are high-dimensional and abstract, capturing features related to patient medical conditions that can be effectively processed only through data-driven AI approaches. Consequently, the proposed integration based on Bayes’ Theorem can offset the limitations of each approach. A schematic of the overall concept of PIML is shown in Figure 1, illustrating how prior knowledge, clinical reasoning, and machine learning interact to enhance final diagnostic performance.

**FIGURE 1:**
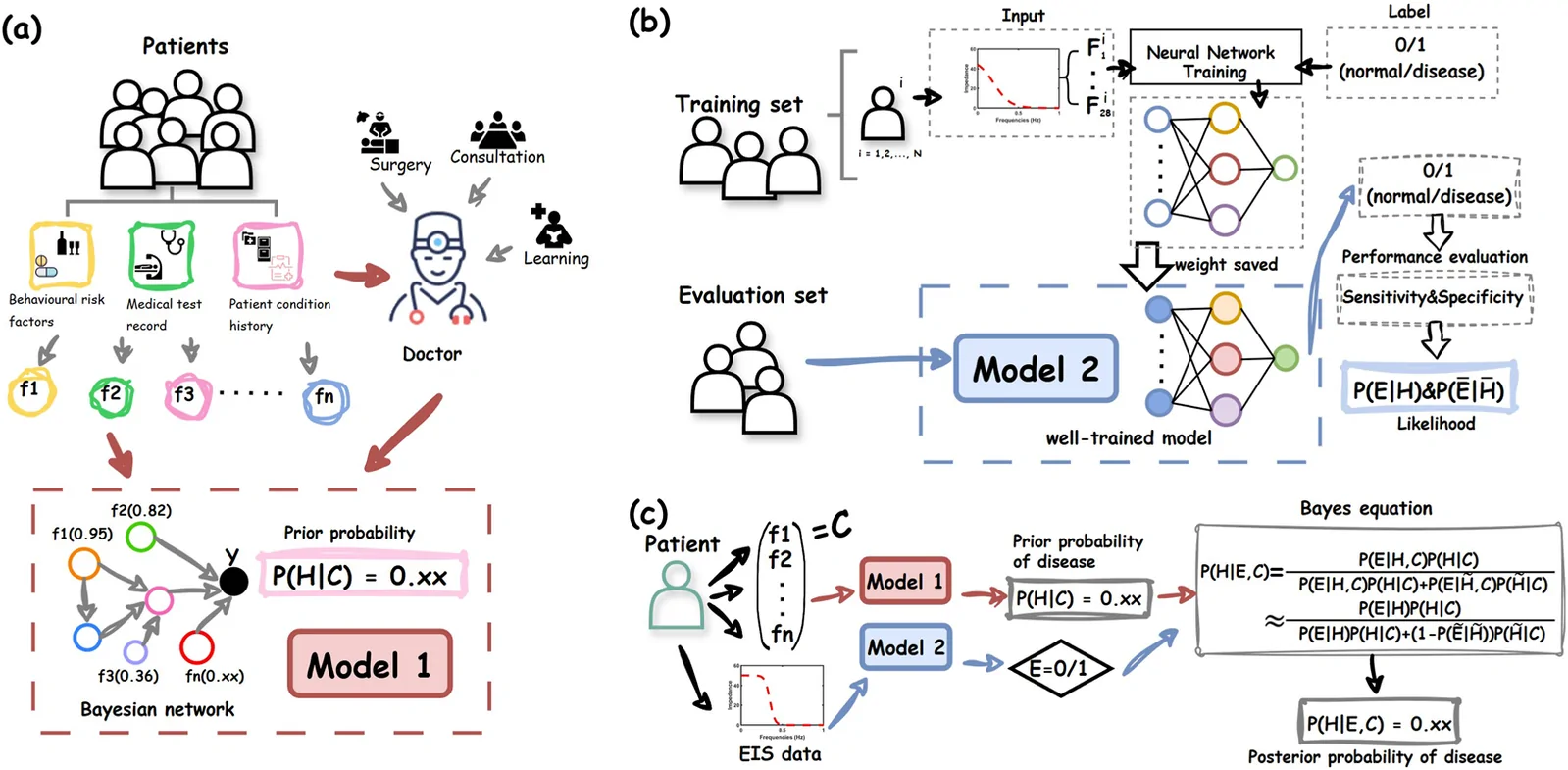
Conceptual overview of the proposed PIML (Prior-informed Machine Learning). **(a)** Building Model 1: Model 1 represents the PIML framework’s approach to determining the prior probability, which follows the general principles of the Bayesian network. Specifically, following this general principle, the patient-specific prior probability may be obtained from clinically relevant sources such as empirical clinical statistics, patient history, epidemiological or institutional evidence, validated clinical risk tools, expert assessment, or probabilistic models. In the present study, different prior-generation strategies were intentionally used across three case studies to demonstrate the flexibility of the proposed approach. Given a new patient’s observed factors at admission, Model 1 can perform probabilistic inference to produce the probability of disease, which is used as the prior for downstream analysis. **(b)** In this part, high-dimensional data, such as, e.g., EIS data in our study, are used to train an ML model for the diagnosis of the same disease. The 1 × 28 representation shown in the figure is a schematic illustration of the raw EIS data, consisting of the real and imaginary impedance measurements acquired at 14 frequencies. In the actual experimental pipeline, these raw EIS measurements are further processed according to the specific downstream task. As described in the Methods, the high-risk oral lesion detection and preterm birth datasets are represented by four Cole-model parameters, whereas the cervical cancer dataset is represented by an eight-dimensional feature vector derived from the mean and range of the four Cole parameters across multiple spatial readings. These processed features are then used to train Model 2. Once training is completed, the model parameters (e.g., weights). are saved, and the well-trained ML model with an optimized performance in terms of sensitivity and specificity is referred to as Model 2. Then, an independent evaluation dataset is subsequently used to validate the model, providing evidence that the optimized sensitivity and specificity are expected to be attained in real-world clinical practice. **(c)** When Model 1 and Model 2 are applied in clinical practice, the patient-specific information and factors C=(f1, f2…fn)^T^ are first processed through Model 1 to obtain the prior probability of disease occurrence. Subsequently, the high-dimensional data collected from the patient, such as, e.g., the EIS data, are input into Model 2 to generate an ML-enabled evidence-based diagnosis result (E = 0 or 1). Finally, the prior probability from Model 1, the evidence-based diagnosis outcome from ML Model 2, and the performance of Model 2 in terms of sensitivity and specificity are combined using the Bayesian inference, yielding a posterior probability that incorporates both prior clinical knowledge and data-driven evidence to produce a Bayesian-updated diagnostic estimate.

The proposed PIML framework could leverage the rapid, high-level feature extraction and complicated features-based classification capabilities of ML models while preserving the indispensable role of human clinical judgement in the decision-making process. Its key contributions are:

1. The proposed PIML framework integrates ML-derived diagnostic predictions with patient-specific clinical priors. By treating ML predictions as evidence-based diagnostic information and combining them with patient-specific prior probabilities through Bayes’ Theorem, PIML generates interpretable posterior probabilities of disease. This approach mitigates the limited generalizability of ML-only diagnostic models and enhances their applicability in clinical settings.
2. PIML integrates the determination of sensitivity and specificity required for Bayesian inference into an end-to-end ML model development process. By optimizing classification thresholds during ML model development and validation, the PIML framework systematically links the predictive performance of the ML model with subsequent Bayesian updating. This provides a coherent mechanism for combining ML-based discriminative prediction with clinical information-based generative diagnosis, with potential applications across a wide range of medical diagnostic challenges.
3. This study evaluates the proposed PIML framework across three representative diagnostic tasks (cervical neoplasia detection, high-risk oral lesion detection and preterm birth) in which all machine learning models were developed using electrical impedance spectroscopy (EIS) datasets. Overall, the results demonstrate that PIML improves diagnostic performance over ML-only approaches and, in most dataset–classifier combinations, outperforms prior-only and feature-level prior integration strategies.

Together, these contributions establish a principled and systematic framework for integrating ML-derived diagnostic evidence with clinically relevant prior information, advancing the development of more interpretable, clinically grounded, and reliable medical diagnosis.

## 2 Materials and methods

### 2.1 Methods

#### 2.1.1 EIS data and bio-impedance spectrum curve fitting

Electrical impedance spectroscopy (EIS) is a non-invasive modality that characterizes tissue properties by measuring its impedance across a range of frequencies. In this study, we collected multi-frequency EIS signals from three independent clinical datasets. Each EIS reading comprises complex impedance values measured at 14 distinct frequencies ranging from 76 Hz to 625 kHz and can be represented by two components (Buendia et al., 2011): the real part $Z_{Re}$ and the imaginary part $Z_{Im}$ as follows (Equation 1):

$$Z\left(\omega \right)=Z_{Re}\left(\omega \right)-jZ_{Im}\left(\omega \right)$$ (1)

Each dataset was composed of 14 real and 14 imaginary parts.

To extract physiologically meaningful features from raw EIS data, we employ the Cole model, a well-established circuit-based representation of bio-impedance. The Cole model is used to describe the relationship between the measured tissue impedance $Z\left(\omega \right)$ and frequency f , commonly represented by the following form (Equation 2):

$$Z\left(f\right)=R_{\infty }+\frac{R_{0}-R_{\infty }}{1+{\left(\frac{jf}{f_{c}}\right)}^{1-\alpha }}$$ (2)

where $R_{0}$ and $R_{\infty }$ represent the resistances at zero and infinite frequencies, respectively, and primarily determine the impedance spectrum values at the low- and high-frequency ends of the measurement range. f is the frequency of excitation current used in EIS data measurement and from 76 Hz to 625 kHz. $f_{c}$ is referred to as the characteristic frequency and *α* is α constant ( 0≤α≤1 ). These four Cole model parameters $R_{0}$ , $R_{\infty }$ , $f_{c}$ and *α* are associated with the properties of the biological tissue. To estimate these parameters from the measured EIS data, we used non-linear curve fitting in MATLAB (MATLAB, 2022a) using the trust-region-reflective algorithm implemented in the ‘lsqcurvefit’ function. The objective was to minimize the squared error between the measured and modelled impedance magnitudes over the entire frequency range.

As shown in Figure 2, the Cole model achieves a close approximation to both the real and imaginary parts of the measured impedance data across the frequency spectrum.

**FIGURE 2:**
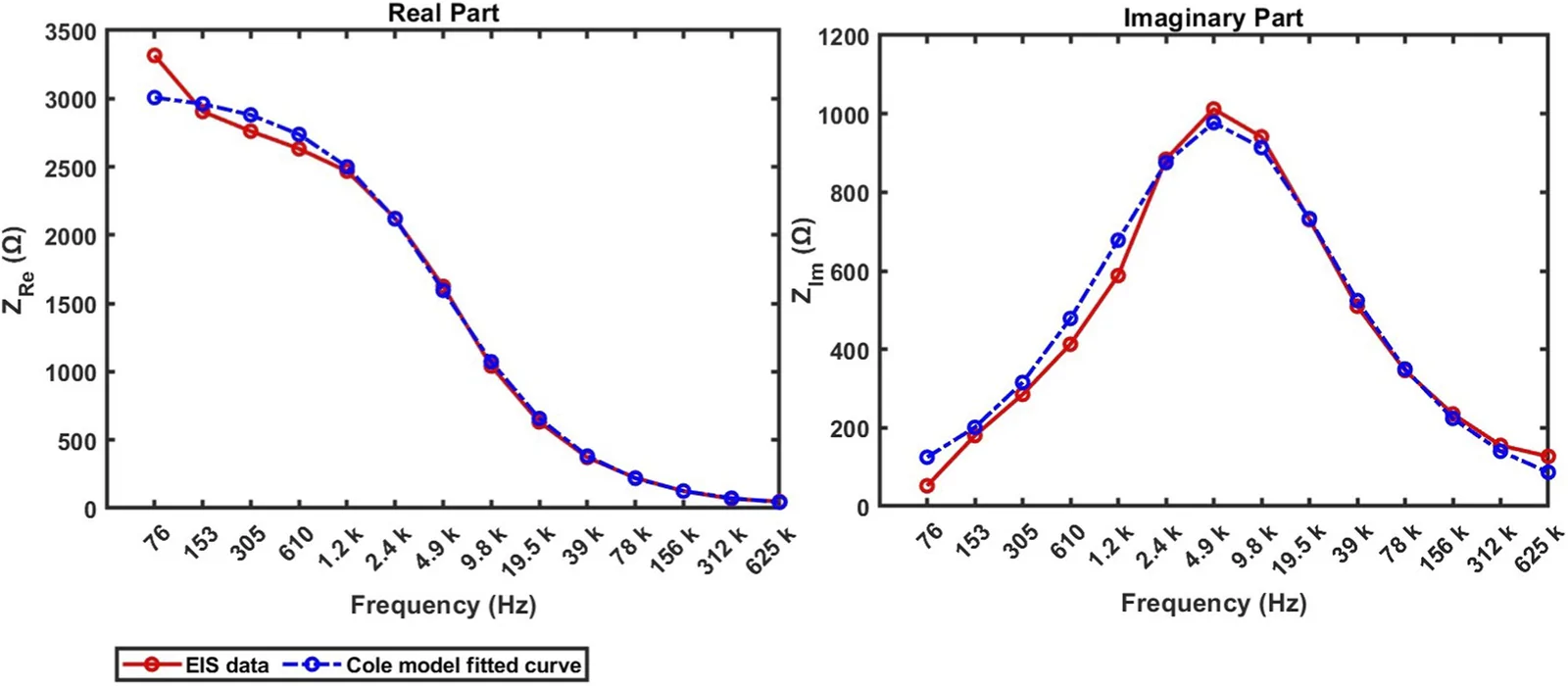
Representative fitting results of the Cole model to EIS data. The left and right panels respectively show the real and imaginary parts of the impedance spectrum across chosen 14 frequency points. Red curves represent the measured EIS data, and blue curves represent the fitted Cole model curve.

#### 2.1.2 Hyperparameter tuning via AUC maximization

To achieve optimal predictive configuration, we used grid search over each model’s hyperparameter space. For each candidate set of hyperparameters θ∈Θ , the corresponding model $M_{\theta }$ is trained on $D_{train}$ and validated on $D_{vali}$ . The area under the receiver operating characteristic curve (AUC) in validation results is used as the model selection metric. The best-performing configuration is determined as: $\theta^{*}=\arg\max_{\theta \in \Theta }{AUC}_{val}\left(M_{\theta }\right)$ . The ${AUC}_{val}$ compared here are the average of each model’s performance for each configuration, obtained after repeating 100 times. Once optimal hyperparameters are selected, the final model $M_{\theta^{*}}$ is fixed for next inference and evaluation stages.

#### 2.1.3 Machine learning models

Four widely adopted classification models are selected to assess the robustness and generalizability of our methodology: decision tree (DT), random forest (RF), support vector machine (SVM), and multilayer perceptron (MLP).

DT constructs a hierarchical tree structure where internal nodes represent decision rules based on feature thresholds, and leaf nodes denote class outcomes. Its interpretability and efficiency make it suitable for preliminary pattern recognition in structured medical data. In hyperparameter tuning, three key parameters are considered: ‘MaxNumSplits’ (maximum number of binary splits); ‘MinParentSize’ (minimum number of observations required to split a node); ‘MinLeafSize’ (minimum number of observations required at a leaf node). Each parameter was tuned over the discrete set {5,10,15,20,25,30}, resulting in a total of 216 combinations.

RF is an ensemble of decision trees trained on bootstrapped subsets of the data with random feature selection at each split. This model mitigates overfitting and improves predictive stability by averaging outputs across multiple trees. The final predicted probability is computed by averaging the outputs of individual trees: ${\hat{p}}_{j}=\frac{1}{T}\sum_{t=1}^{T}{\hat{p}}_{j}^{\left(t\right)}$ where T denotes the number of trees, and ${\hat{p}}_{j}^{\left(t\right)}$ is the prediction from the t-th tree. In hyperparameter tuning, three key parameters are considered: ‘NumTrees’ (total number of trees): {20,40,60,80,100,120}; ‘MinLeafSize’ and ‘MaxNumSplits’: {5,10,15,20,25,30}.

SVM constructs a decision boundary that maximizes the margin between support vectors in a (possibly transformed) feature space. In this study, we considered both linear and non-linear kernels to capture different data structures. Specifically, we employed: the linear kernel $K\left(x_{i},x_{j}\right)=x_{i}^{T}x_{j}$ and the radial basis function (RBF) kernel: $K\left(x_{i},x_{j}\right)=\exp\left(-\gamma {\left\|x_{i}-x_{j}\right\|}^{2}\right)$ . The choice of kernel function allows the model to adapt to both linearly separable and non-linearly separable data. In hyperparameter tuning, two key parameters are considered: ‘KernelFunction’: {‘linear’, ‘rbf’}; ‘BoxConstraint’: {0.0001,0.001,0.01,0.1,1,10,100}, which controls the penalty for misclassification.

MLP is a feedforward neural network that learns complex non-linear mappings by composing multiple affine transformations and activation functions. It maps input features $x_{j}\in R^{d}$ to an output probability ${\hat{p}}_{j}\in \left[0,1\right]$ via: ${\hat{p}}_{j}=\sigma \left(W^{\left(L\right)}f^{\left(L-1\right)}\left(\ldots f^{\left(1\right)}\left(W^{\left(1\right)}x_{j}+b^{\left(1\right)}\right)\ldots \right)+b^{\left(L\right)}\right)$ , where $f^{\left(l\right)}$ is a non-linear activation function (ReLU in this study), L is the number of hidden layers, and $\sigma \left(.\right)$ denotes the sigmoid output activation for binary classification. Considering the limited dataset, we performed grid search over lightweight model structure with one or two hidden layers, with following configuration {number of units in layers: ([10], [20], [30], [10,10], [20,10], [30,20])}. All models were trained using binary cross-entropy loss via Adam and default learning rate (MATLAB, 2022a).

The optimal model architecture for each classifier was selected based on the maximum area under the ROC curve (AUC) achieved on the validation set during hyperparameter tuning. The complete list of selected hyperparameter configurations across all models and datasets is provided in the Supplementary Table S3.

#### 2.1.4 Initial threshold-based classification

After tuning stage, the model is retrained on $D_{train}$ and applied to both $D_{vali}$ and $D_{test}$ to produce predicted scores (probabilities) for each class (Equations 3, 4):

$${\hat{p_{i}}}^{val}=M_{\theta^{*}}\left(x_{i}\right),x_{i}\in D_{vali}$$ (3)

$${\hat{p_{i}}}^{test}=M_{\theta^{*}}\left(x_{j}\right),x_{j}\in D_{test}$$ (4)

Using our previously published threshold selection method $TS\left(\cdot \right)$ (Lin et al., 2026), the optimal classification threshold is computed on the predicted scores ${\hat{p_{i}}}^{val}$ (Equation 5):

$$\tau_{1}=TS\left({\hat{p_{i}}}^{val}\right)$$ (5)

Following the threshold selection strategy, the classification threshold was determined from the ROC curve of the validation data rather than using the default probability threshold of 0.5. Briefly, an initial threshold was identified according to the Youden criterion, which considers both sensitivity and specificity. Because diagnostic applications may require greater emphasis on sensitivity, particularly for identifying disease-positive cases, the threshold was further evaluated by progressively reducing the Youden-derived value. For each candidate threshold, the corresponding sensitivity and specificity were calculated on the validation set, and the final threshold was selected to achieve an appropriate balance between improved sensitivity and acceptable specificity. In the present study, the final threshold was defined as the first candidate threshold achieving a sensitivity of at least 70% while maintaining a specificity of at least 50% on the validation set. If no candidate threshold simultaneously satisfied both criteria, the threshold with the highest sensitivity among those maintaining a specificity of at least 50% was selected. Once selected, the threshold was fixed and applied directly to the test set without further adjustment. These operating criteria can be pre-specified differently for other clinical applications according to their relative requirements for sensitivity and specificity. Candidate thresholds were expressed as $T=\alpha T_{Y}$ , where $T_{Y}$ denotes the Youden-derived threshold. The scaling factor α was sequentially decreased from 1.0 to 0.1 in fixed increments of 0.1, i.e., α = 1.0, 0.9, 0.8 … 0.1, to progressively lower the classification threshold while monitoring the corresponding sensitivity and specificity on the validation set. Once determined, this threshold was fixed and subsequently applied to the test-set prediction scores. No information from the test set was used during threshold selection.

Then, the binary predictions are calculated as Equation 6:

$${\hat{y}}_{j}^{\left(1\right)}=\left\{\begin{array}{c} 1\;if\;{\hat{p_{j}}}^{test}\geq \tau_{1}\\ 0\;otherwise \end{array}\right.$$ (6)

The sensitivity ( $S_{e}^{val}$ ) and specificity ( $S_{p}^{val}$ ) on the validation set are also computed using threshold $\tau_{1}$ for subsequent steps.

#### 2.1.5 Bayesian posterior correction

We incorporate domain knowledge via class-conditional prior-based Bayesian inference. From the training set $D_{train}$ , the prior probability can be estimated as the probability of disease given the available clinical information C such that Equation 7:

$$P\left(H|C=c\right)=\frac{\sum_{i\in D_{train}}1\left(C_{i}=c\right)y_{i}}{\sum_{i\in D_{train}}1\left(C_{i}=c\right)}$$ (7)

where $1\left(\cdot \right)$ is an indicator function and $C_{i}$ represents the clinical information of the i^th^ patient. For simplicity, this quantity is referred to as the “prior probability” throughout the remainder of the manuscript. When applying the PIML framework, it is assumed that $P\left(E|H,C\right)=P\left(E|H\right)$ and $P\left(E|\tilde{H},C\right)=P\left(E|\tilde{H}\right)$ , where E denotes the ML-derived diagnostic evidence. This assumption allows the sensitivity and specificity of the ML model, as estimated from the validation data, to be directly incorporated into the subsequent Bayesian updating procedure. The assumption may be clinically justifiable when the available clinical information (C) and the ML/EIS evidence (E) capture complementary and distinct aspects of the disease of concern, including its manifestations and related factors. For instance, in the cervical application, this assumption can be considered clinically plausible. In this case, EIS, colposcopy, and cytology characterize different aspects of cervical pathology: EIS measures tissue electrical properties, colposcopy evaluates macroscopic visual abnormalities, and cytology assesses cellular morphological changes. Accordingly, previous clinical studies have investigated EIS as an adjunct to colposcopy and cytology, demonstrating that the combined use of EIS, colposcopy, and cytology can improve diagnostic outcomes (Tidy et al., 2013). This complementarity supports the rationale for treating EIS-derived evidence as a distinct source of diagnostic information, although it does not constitute formal statistical proof of conditional independence.

Then, by applying the Bayesian Theorem, the posterior probability of each subject can be determined as Equations 8, 9:

$$\begin{array}{c} P\left(H|E,C\right)=\frac{P\left(E|H,C\right)P\left(H|C\right)}{P\left(E|C\right)}=\frac{P\left(E|H,C\right)P\left(H|C\right)}{P\left(E|H,C\right)P\left(H|C\right)+P\left(E|\tilde{H},C\right)P\left(\tilde{H}|C\right)}\\ \approx \frac{P\left(E|H\right)P\left(H|C\right)}{P\left(E|H\right)P\left(H|C\right)+P\left(E|\tilde{H}\right)P\left(\tilde{H}|C\right)} \end{array}$$ (8)

$$\begin{array}{c} P\left(H|\tilde{E},C\right)=\frac{P\left(\tilde{E}|H,C\right)P\left(H|C\right)}{P\left(\tilde{E}\left|C\right.\right)}=\frac{P\left(\tilde{E}|H,C\right)P\left(H|C\right)}{P\left(\tilde{E}|H,C\right)P\left(H|C\right)+P\left(\tilde{E}|\tilde{H},C\right)P\left(\tilde{H}|C\right)}\\ \approx \frac{P\left(\tilde{E}|H\right)P\left(H|C\right)}{P\left(\tilde{E}|H\right)P\left(H|C\right)+P\left(\tilde{E}|\tilde{H}\right)P\left(\tilde{H}|C\right)} \end{array}$$ (9)

where $P\left(E|H\right)$ , $P\left(E|\tilde{H}\right),P\left(\tilde{E}|H\right),\text{and }P\left(\tilde{E}|\tilde{H}\right)$ correspond to the true-positive rate (TPR, sensitivity), false-positive rate (FPR, 1-specificity), false-negative rate (FNR, 1-sensitivity), and true-negative rate (TNR, specificity) of the machine-learning model, respectively.

$\text{Here},P\left(H|E,C\right)$ represents the Bayesian-updated probability estimate of disease under the stated assumptions when the model predicts a positive result while $P\left(H|\tilde{E},C\right)$ represents the Bayesian-updated probability estimate of disease under the stated assumptions when the model predicts a negative result.

We use the sensitivity and specificity obtained from the validation data ( $S_{e}^{val}$ and $S_{p}^{val}$ ) to determine $P\left(E|H\right)$ , $P\left(E|\tilde{H}\right),P\left(\tilde{E}|H\right),$ and $P\left(\tilde{E}|\tilde{H}\right)$ in Equations 8, 9. The two equations are then applied to both the test and validation data to obtain the posterior probability ${\tilde{p}}^{test}$ and ${\tilde{p}}^{val}$ , respectively. The details can be found in Supplementary Figure S2.

#### 2.1.6 Recalibration via posterior thresholding

The Bayesian-corrected probabilities on the validation data ${\tilde{p}}^{val}$ are input to the threshold selection module again to determine the second decision boundary (Equation 10):

$$\tau_{2}=TS\left({\tilde{p}}^{val}\right)$$ (10)

Then, final predictions on the test set are made by applying this threshold $\tau_{2}$ to the posterior (Equation 11):

$${\hat{y}}_{j}^{\left(2\right)}=\left\{\begin{array}{c} 1\;if\;{\tilde{p}}_{j}^{test}\geq \tau_{2}\\ 0\;otherwise \end{array}\right.$$ (11)

#### 2.1.7 Performance evaluation and statistical significance

To avoid model uncertainty, all steps—including data splitting, model training, thresholding, and Bayesian adjustment—are repeated 100 times with different random seeds (MATLAB, 2022a). For each iteration, we record the test AUC, sensitivity, and specificity before and after Bayesian correction step. For each pair of compared results (model or method), the differences in individual performance metrics were first evaluated for normality using the Kolmogorov–Smirnov test. Given that the assumption of normality was violated, statistical comparisons were performed using the Wilcoxon signed-rank test, a non-parametric alternative suitable for paired data (Lin et al., 2024). Because all compared methods were evaluated using identical data partitions within each repetition, statistical comparisons were performed in a paired manner using the Wilcoxon signed-rank test. This paired analysis reduces the influence of between-partition variability when comparing different methods, because method-wise performance differences are evaluated under identical patient compositions within each repetition. We note, however, that the repeated random partitions are not statistically independent because individual subjects may occur in multiple repetitions. Accordingly, the resulting p-values and uncertainty intervals should be interpreted as reflecting the consistency of method-wise differences across repeated partitions rather than as evidence derived from 100 independent patient cohorts. Performance gains are reported as relative percentage increases along with statistical significance levels (e.g., * denotes p<0.05 , ** denotes p<0.01 ).

For ROC analysis, AUC was calculated from prediction scores before the final binary decision threshold was applied. Specifically, baseline ML AUC was calculated from the original continuous test prediction scores before application of $\tau_{1}$ ; prior-only AUC was calculated from the patient-specific prior probabilities; and ML + prior AUC was calculated from the continuous prediction scores generated by the corresponding classifier. For PIML and PIML (APP), ROC curves and AUC values were calculated from the Bayesian-updated posterior probability scores before application of $\tau_{2}$ . $\tau_{1}$ and $\tau_{2}$ were therefore used to define diagnostic operating points for the Bayesian update and final sensitivity/specificity calculation, respectively. The 100 repeated experiments were performed using MATLAB random seeds from 1 to 100. For visualization purposes, the ROC curves shown in Figures 3–5 were consistently generated from the repetition corresponding to random seed = 25. The same seed was used for all compared methods within each task, while all quantitative results reported in the tables were calculated across the full 100 repetitions.

**FIGURE 3:**
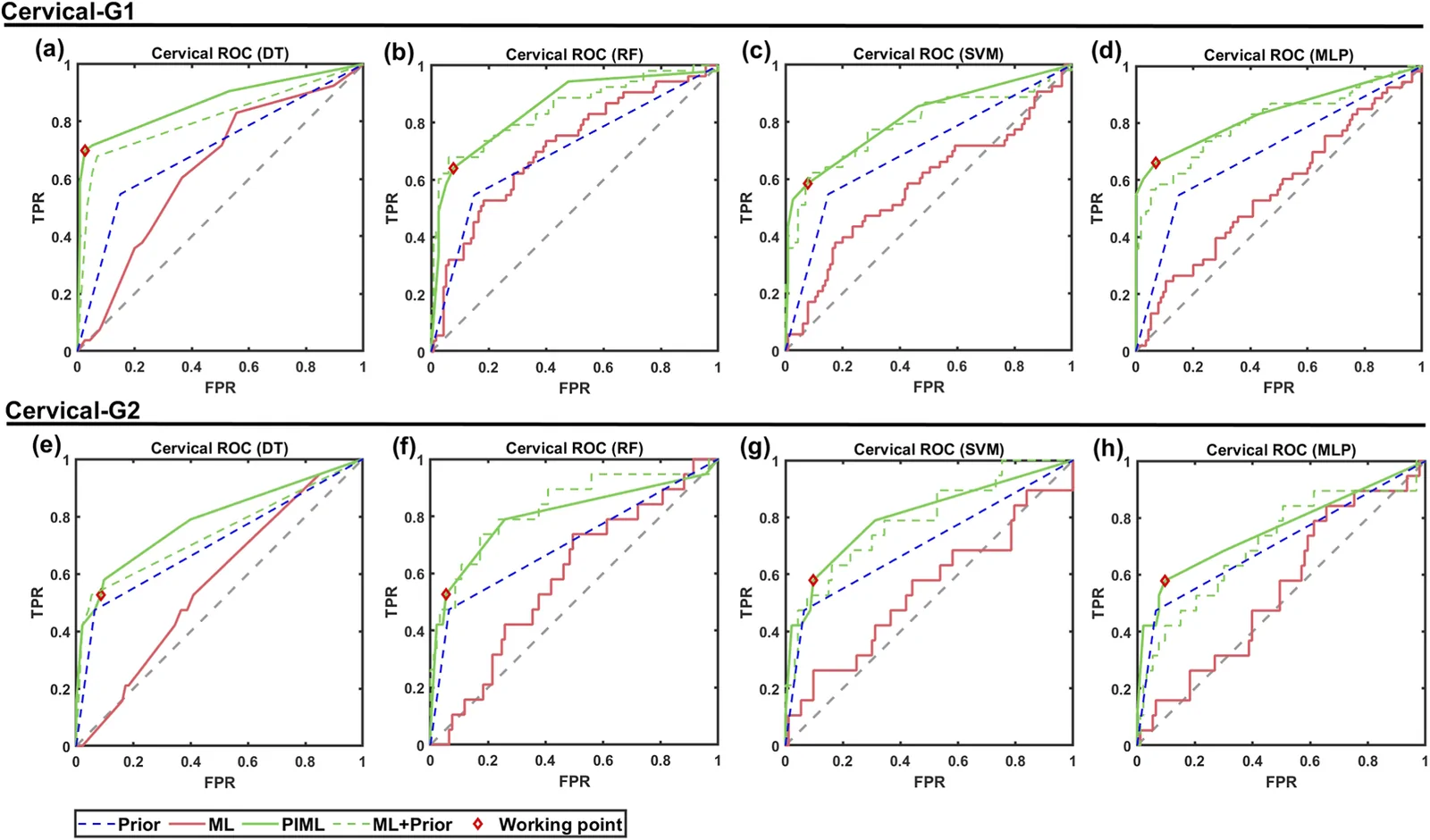
Performance comparison on cervical neoplasia detection using different methods. **(a–d)** Receiver operating characteristic (ROC) curves on the Cervical-G1 dataset for four machine learning models: decision tree (DT), random forest (RF), support vector machine (SVM), and multilayer perceptron (MLP). **(e–h)** Corresponding results on the Cervical-G2 external test set. In each subplot, the blue dashed line represents the prior-only based performance, and the red solid line represents baseline ML performance without adding any prior knowledge. The green dashed line represents a control method in which prior knowledge (colposcopy and smear test) is directly concatenated with the input features. The green solid line represents our proposed PIML method integrating priors via Bayesian inference. The ROC curves illustrate that both PIML and ML + prior generally improve discrimination relative to baseline ML, although their relative performance varies across classifiers. In particular, different from other cases where PIML performed better than ML + prior in terms of AUC, ML + prior achieved a higher AUC than PIML for the RF classifier in both Cervical-G1 and Cervical-G2, indicating a classifier-dependent performance with the two prior-integration strategies. Red diamond markers indicate the operating points determined from the validation-derived thresholds (Lin et al., 2026). The ROC curves shown correspond to one predefined repetition (MATLAB random seed = 25); aggregate quantitative performance across all 100 repetitions is reported in the corresponding table.

**FIGURE 4:**
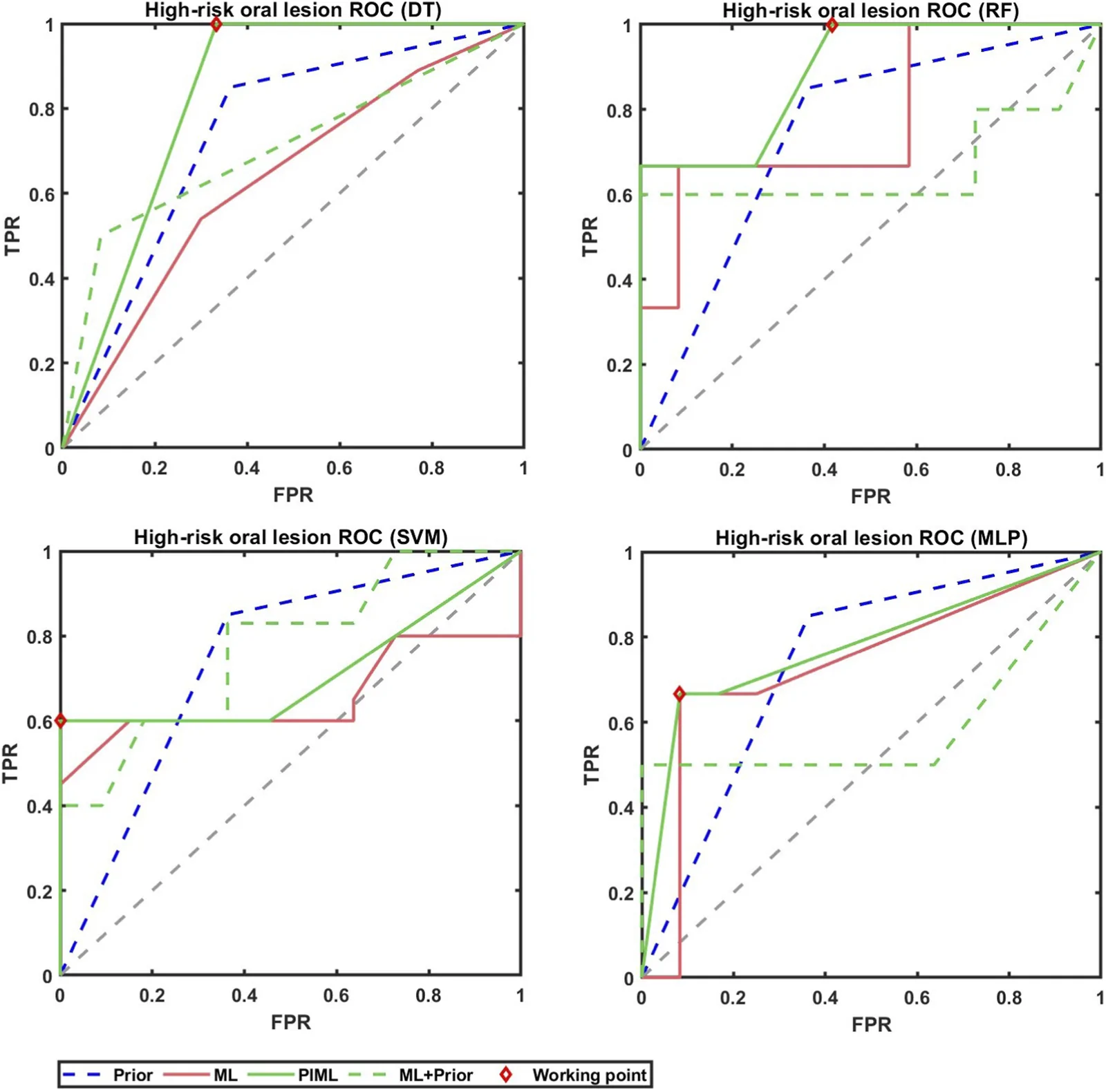
Performance comparison on high-risk oral lesion detection using different methods. Receiver operating characteristic (ROC) curves on high-risk oral lesion dataset for four machine learning models: decision tree (DT), random forest (RF), support vector machine (SVM), and multilayer perceptron (MLP). In each subplot, blue dashed line represents the prior-only based performance, red solid line represents baseline ML performance without adding any prior knowledge. Green dashed line represents a control method where prior knowledge (historical record of smoking) is directly concatenated with input features. Green solid line represents our proposed PIML method integrating priors via Bayesian inference. The ROC curves shown correspond to one predefined repetition (MATLAB random seed = 25); aggregate quantitative performance across all 100 repetitions is reported in the corresponding table.

**FIGURE 5:**
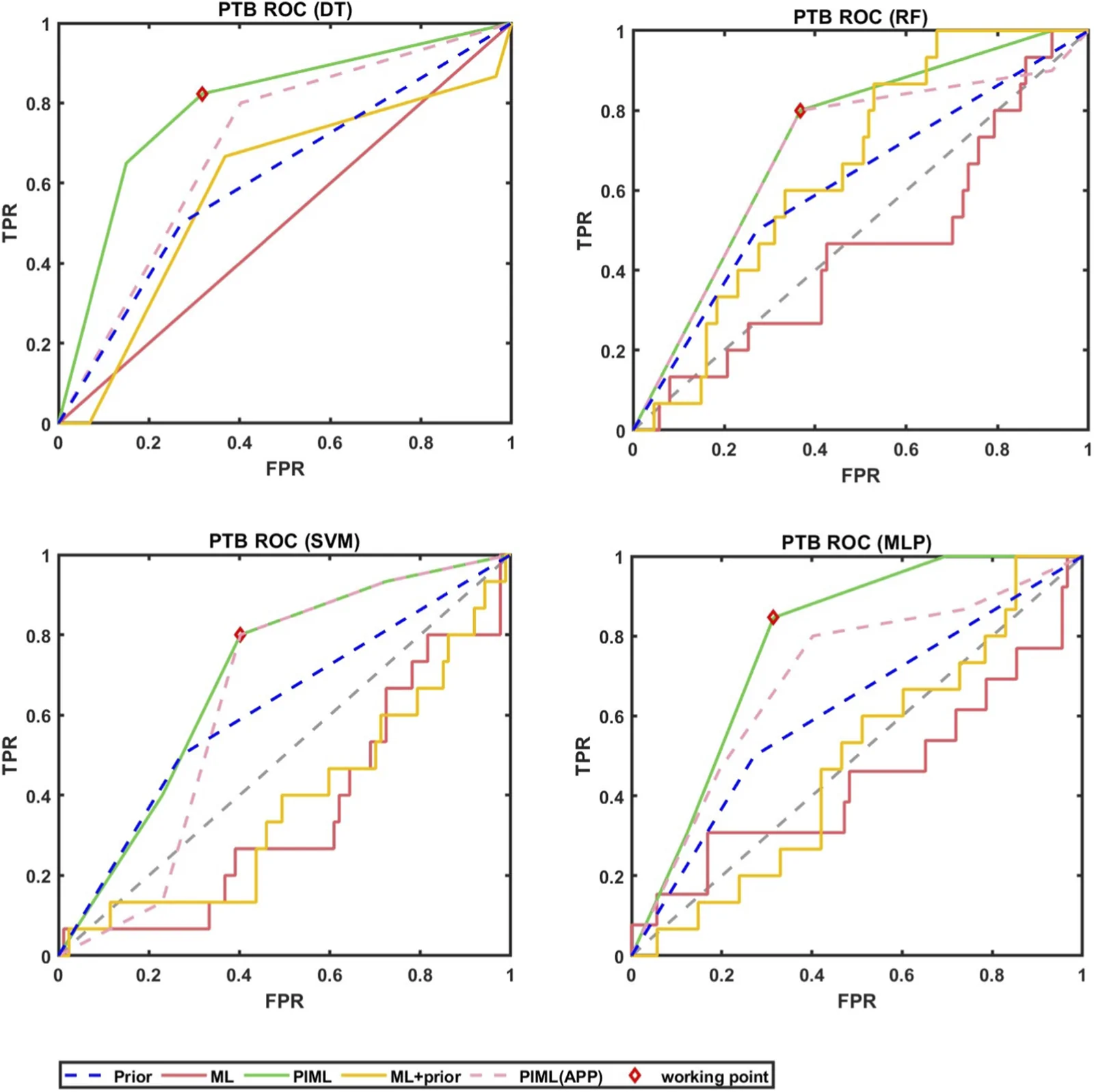
Performance comparison on PTB diagnosis using different methods. ROC curves for preterm birth prediction across four machine learning models: DT, RF, SVM and MLP. Five methods are compared: prior-only based method (blue dashed line), baseline ML without prior knowledge (red solid line), ML with direct concatenation of prior feature (historical preterm birth record) (yellow solid line), our proposed PIML method via Bayesian inference (green solid line), and PIML using externally derived prior from the QUiPP app (pink dashed line). Red diamond markers indicate the selected working points based on validation set threshold optimization. The ROC curves shown correspond to one predefined repetition (MATLAB random seed = 25); aggregate quantitative performance across all 100 repetitions is reported in the corresponding table. Both PIML and PIML (APP) show substantially stronger discrimination than the corresponding baseline ML models and generally provide a more balanced sensitivity-specificity profile. The similar behaviour of PIML and PIML (APP) further indicates that the framework can accommodate both internally estimated and externally derived clinical priors.

### 2.2 Dataset

#### 2.2.1 Cervical cancer dataset

This study utilized a comprehensive cervical cancer dataset from previous research (Brown et al., 2005; Brown et al., 2000), comprising two independent cohorts with consistent diagnostic modalities (Tidy et al., 2013).

The primary cohort consists of 1677 samples (Cervical-G1), collected during routine cervical screening, with 528 biopsy-confirmed cases of cervical intraepithelial neoplasia or carcinoma, and 1149 normal samples. For each subject, three complementary diagnostic sources were collected: (a) Electrical Impedance Spectroscopy (EIS) signals obtained using an EIS device ZedScanTM (Zilico Limited, UK), which provides quantitative measurements of cervical epithelial electrical properties during real-time colposcopy. EIS spectral measurements are made at up to 12 reading sites around cervix (minimum number of sites is 8) of individual women (Tidy et al., 2013). (b) Colposcopic visual assessments, recorded by experienced clinicians using standard acetic acid application protocols. (c) Cytological smear test results (Papanicolaou smear), consistent with standard gynaecological screening practice.

The second cohort of 112 samples (Cervical-G2) was collected from Royal Free Hospital (London, UK) (Tidy et al., 2013), comprising 19 pathologically confirmed cancer cases and 93 normal cases. This cohort followed the same acquisition protocol as the primary dataset and was used to assess the cross-institutional generalizability of chosen machine learning models. Histopathological examination following biopsy served as the ground truth label for all samples, enabling supervised learning and quantitative evaluation.

#### 2.2.2 Oral high-risk lesion dataset

The second dataset employed a non-invasive high-risk oral lesion detection dataset from our pilot studies (Murdoch et al., 2014; Lin et al., 2026) comprising EIS signals collected from anatomically standardized oral cavity subsites. A total of 62 subjects were enrolled and classified based on clinical diagnosis into low or high risk groups following a binary classification standard (Lin et al., 2026), including 53 normal subjects, with no clinically significant lesions and 9 high-risk subjects, presenting with histopathologically confirmed oral epithelial dysplasia or malignant oral lesions.

EIS measurements were collected by the same device ZedScan^TM^, with data acquired from up to 12 intraoral subsites per subject, including the floor of mouth (FM) and ventral tongue (VT). Reading from these two sites were used together in analysis based on clinical consistency validated by dental clinicians to compensate the data insufficiency.

For healthy cases, two readings (from FM and VT) were achieved per subject, yielding 100 readings in total. For high-risk cases, 1 to 3 EIS readings (from FM or VT) were achieved per subject based on lesion location, yielding 18 high-risk readings. Given the class imbalance and the limited number of high-risk subjects, stratified random splitting was performed at the subject level according to the diagnostic label. All EIS readings obtained from the same subject were assigned exclusively to a single subset according to patient ID, thereby preventing subject-level information leakage between the training, validation, and test data. As each of the 9 high-risk subjects contributed between 1 and 3 EIS readings, the splitting procedure was additionally constrained to ensure that both the validation and test sets contained at least 3 high-risk EIS readings in every repetition.

In addition to impedance measurements, self-reported smoking status and historical record was collected for each subject during demographics and questionnaires (Murdoch et al., 2014). Smoking history was treated as a categorical risk factor. In statistical treatment, individuals with a history of long-term smoking exceeding 10 years (classified as ex-smokers) were grouped with current smokers. Within the high-risk group, two subjects fell into current smokers.

#### 2.2.3 Preterm birth dataset

The third dataset includes a clinical dataset for early detection of preterm birth (PTB), collected as part of a pilot study conducted at the University of Sheffield (UK) (Tian et al., 2023). The dataset comprises a total of 438 pregnant subjects, each undergoing non-invasive cervical assessment using electrical impedance spectroscopy (EIS) at mid-gestation. Based on the gestational outcome, subjects were categorized into preterm birth (PTB) cases (delivery before 37 gestational weeks; n = 62) and term deliveries (n = 376), following standard obstetric definitions.

Each subject contributed between 1 and 3 EIS readings per clinical visit, depending on data quality and operator judgment, resulting in a total of 1,022 cervical EIS measurements. The EIS signals were collected using a dedicated vaginal probe (the Sheffield Mark 5.0 device (Tian et al., 2023)) under standard clinical conditions, targeting the cervical epithelium. All data were labeled postnatally based on the recorded gestational age at delivery.

In addition to EIS measurements, clinical background information was collected through patient self-report and medical records, including known PTB history. This history record was used as a domain-informed prior to support model reasoning under a Bayesian formalism. To prevent information leakage, all training and evaluation readings were conducted at the subject level, such that no patient contributed samples to both training and test sets.

A summary of all datasets is provided in Table 1. For the cervical cancer dataset, each subject contributed 8 to 12 EIS signal recordings obtained from distinct locations on the cervix. In both the table and experimental design, a single representative reading per subject was used, computed as either the mean or differential value across all intra-subject measurements.

**TABLE 1:** Summary of dataset characteristics.

Dataset	Subjects	Readings	Annotation type	Prior knowledge
Cervical	1677 + 112	1677 + 112	Biopsy	Colposcopy + smear test
Oral	62	119	Histopathology	Smoking history
Preterm	438	1022	Delivery gestational age	History of preterm birth

#### 2.2.4 Data pre-processing

For all datasets, raw EIS signals were transformed into parametric representations using the non-linear Cole model (Tidy et al., 2013), yielding four key parameters, $R_{0}$ , $R_{\infty }$ , $f_{c}$ and *α*. For the high-risk oral lesion detection and preterm birth (PTB) datasets, each EIS reading was thus directly mapped to one 4-dimensional feature vector (Equation 12):

$$x=\left[R_{0},R_{\infty },f_{c},\alpha \right].$$ (12)

In contrast, the cervical cancer dataset includes multiple spatial readings per subject (8–12), recorded from distinct locations around the cervix. To capture the spatial heterogeneity, we first calculate the Cole model parameters from each reading i under same subject, then calculate the mean and maximum difference across all readings. For example, for $R_{\infty }$ , the $\Delta R_{\infty }$ and $\bar{R_{\infty }}$ are calculated as following (Equation 13):

$$\Delta R_{\infty }=\max_{i}\left(R_{i}^{\infty }\right)-\min_{i}\left(R_{i}^{\infty }\right),\bar{R_{\infty }}=\frac{1}{n}\sum_{i=1}^{n}R_{i}^{\infty },\left(i=1,2,\ldots ,12\right)$$ (13)

with similar definitions for $R_{0}$ , $f_{c}$ and *α*. Thus, each subject from cervical dataset is represented by an 8-dimensional vector (Equation 14):

$$x_{cervical}=\left[\bar{R_{0}},\bar{R_{\infty }},\bar{f},\bar{\alpha },{\Delta R}_{0},\Delta R_{\infty },{\Delta f}_{c},\Delta \alpha ,\right]$$ (14)

#### 2.2.5 Dataset partitioning and model training

Let the full dataset be denoted as $D={\left\{\left(x_{i},y_{i}\right)\right\}}_{i=1}^{N}$ , where $x_{i}\in R^{d}$ represents the input feature vectors and $y_{i}\in \left\{0,1\right\}$ denotes the ground truth label for our binary classification task. Each of the three clinical cohorts (cervical neoplasia detection, high-risk oral lesion detection, and preterm birth) used in this study is modelled separately. Each dataset is randomly partitioned into training, validation, and test sets in a fixed ratio of 8:1:1, $D\to \left\{D_{train},D_{vali},D_{test}\right\}$ . This procedure is repeated over 100 times with random seeding (MATLAB, 2022a) to control the data variance and ensure repeatability. Each random partition was generated using a predefined random seed to ensure reproducibility. Importantly, within each repetition, exactly the same training, validation, and test sets were used for all methods being compared. Consequently, performance measurements from different methods within the same repetition formed matched observations, ensuring that method-wise comparisons were not confounded by differences in patient composition between methods. The 100 repetitions were used to assess the robustness of model performance to variations in patient-level data partitioning rather than to represent 100 independent patient cohorts or to increase the effective sample size.

For the oral and PTB datasets, dataset partitioning was performed strictly at the subject level, such that all EIS readings from the same patient were assigned exclusively to a single training, validation, or test subset, thereby preventing subject-level information leakage. Model training was performed using individual EIS readings as input observations. However, validation and test performance were evaluated at the patient level. Specifically, prediction scores from repeated EIS readings belonging to the same patient were averaged to obtain a single patient-level score. These aggregated patient-level scores were then used for validation threshold selection and for the calculation of AUC, sensitivity, and specificity. Consequently, each patient contributed only one observation to the validation and test performance evaluation, irrespective of the number of available EIS readings. We employ four representative and widely used machine learning (ML) classifiers: decision tree (DT), random forest (RF), support vector machine (SVM), and multilayer perceptron (MLP). Each model is implemented using standard open-source libraries (MATLAB, 2022a) and trained exclusively on​ $D_{train}$ and $D_{vali}$ during both hyperparameter selection and evaluation.

## 3 Results

### 3.1 Task 1: cervical neoplasia detection

We first evaluated the cervical cancer dataset, comprising two independent cohorts. First group (Cervical-G1) includes 1,677 subjects (528 diagnosed with cervical cancer and 1,149 normal), each with 8–12 electrical impedance spectroscopy (EIS) readings acquired circumferentially around the cervix using a ZedScan^TM^ probe (see first part in Method section). Each reading was fitted using the Cole model (Buendia et al., 2011) to extract four electrical parameters ( $R_{0}$ , $R_{\infty }$ , $f_{c}$ and *α*), from which we derived an 8-dimensional feature vector for each subject composed of the average and range (max–min) of each parameter, yielding an 8-dimensional representation per subject, $x_{cervical}=\left[\bar{R_{0}},\bar{R_{\infty }},\bar{f},\bar{\alpha },{\Delta R}_{0},\Delta R_{\infty },{\Delta f}_{c},\Delta \alpha ,\right]$ . Second group (Cervical-G2), comprising 112 additional cases (19 positive, 93 normal) collected from Royal Free Hospital in London, served as an independent external test set to assess model external generalizability.

Two forms of structured clinical prior knowledge-colposcopy examination and smear test results, were incorporated in this task, both of which reflect established diagnostic protocols and are routinely considered by clinicians. These prior probabilities were encoded as binary variables: $f_{Colp,}f_{Smear}\in \left\{0,1\right\}$ .

During validation and testing, the prior probability $P\left(H|C\right)$ required for Bayesian integration module was estimated from statistics of the training set only. Intuitively, the prior probability $P\left(H|C\right)$ can be interpreted as the estimated probability of disease. For instance, if a patient presents both a positive colposcopic and abnormal smear test result, then $P\left(H|C\right)$ corresponds to the empirical proportion of such patients in the training set who were diagnosed with cervical cancer. For the Cervical-G2 external evaluation, all model-related quantities were determined exclusively using Cervical-G1, including the selected hyperparameters, prior probabilities, validation-derived sensitivity and specificity, the initial classification threshold, and the posterior decision threshold. These quantities were then fixed before evaluation on Cervical-G2, and no further tuning, recalibration, or parameter estimation was performed when applied to the external cohort.

Each machine learning model - decision tree (DT), random forest (RF), support vector machine (SVM), and multilayer perceptron (MLP), was independently trained and evaluated. For robust statistical analysis, all experiments were repeated 100 times, and mean performance metrics are reported across repetitions.

In addition to ML–based approaches, we further evaluated a prior-only baseline, in which diagnosis was made only based on prior probabilities derived from training dataset (using colposcopy test results in Task 1), without incorporating EIS-based ML predictions. This baseline reflects a purely knowledge-driven diagnostic strategy and serves as a reference for assessing the added value of data-driven and hybrid approaches.

In addition to comparing the baseline ML models, the proposed PIML method, and the prior-only-based diagnosis method, we also designed a feature concatenation baseline as control group, in which prior knowledge was directly appended to the input vector before training and denoted as following:

$${\tilde{x}}_{cervical}=\left[\bar{R_{0}},\bar{R_{\infty }},\bar{f},\bar{\alpha },{\Delta R}_{0},\Delta R_{\infty },{\Delta f}_{c},\Delta \alpha ,f_{Colp},f_{Smear}\right].$$

Finally, to assess the individual contributions of each prior knowledge factor, we performed ablation experiments in which either colposcopy or smear test results were excluded from the integration. The corresponding results are presented in Supplementary Tables S1, S2.

Table 2 summarizes the quantitative performance for the cervical neoplasia detection task, comparing four diagnostic strategies: prior-only inference, baseline ML, direct feature-level prior integration (ML + prior), and the proposed PIML framework. Overall, both PIML and ML + prior substantially improved diagnostic performance compared with the baseline ML models. PIML achieved higher AUC than ML + prior for most classifier settings, although ML + prior achieved a higher AUC with the RF classifier in both Cervical-G1 and Cervical-G2. These results indicate that the relative advantage of the two prior-integration strategies varies to some extent with the underlying classifier.

**TABLE 2:** Performance comparison on Cervical-G1/G2 dataset.

*Cervical-G1*				
​	Methods	AUC (95% CI)	Specificity (95% CI)	Sensitivity (95% CI)
Model	Prior	0.751 ± 0.036 [0.743 0.758]	0.631 ± 0.471 [0.536 0.725]	0.681 ± 0.243 [0.638 0.735]
DT	ML	0.637 ± 0.037 [0.630 0.644]	0.505 ± 0.119 [0.481 0.528]	0.705 ± 0.132 [0.679 0.732]
	PIML	0.842 ± 0.032 [0.835 0.848]( ↑ 32%) **	0.946 ± 0.021 [0.942 0.950] **	0.650 ± 0.066 [0.637 0.663] **
	ML + prior	0.819 ± 0.037 [0.811 0.826]( ↑ 29%) **	0.934 ± 0.031 [0.928 0.940] **	0.652 ± 0.061 [0.640 0.665] **
RF	ML	0.679 ± 0.036 [0.672 0.686]	0.541 ± 0.121 [0.517 0.565]	0.704 ± 0.132 [0.678 0.730]
	PIML	0.846 ± 0.033 [0.840 0.853]( ↑ 26%) **	0.945 ± 0.040 [0.937 0.953] **	0.645 ± 0.073 [0.631 0.660] **
	ML + prior	0.859 ± 0.033 [0.852 0.865]( ↑ 27%) **	0.924 ± 0.056 [0.913 0.935] **	0.657 ± 0.073 [0.643 0.672] **
SVM	ML	0.638 ± 0.037 [0.630 0.645]	0.657 ± 0.113 [0.635 0.679]	0.560 ± 0.126 [0.535 0.585]
	PIML	0.835 ± 0.041 [0.827 0.844]( ↑ 31%) **	0.912 ± 0.149 [0.883 0.942] **	0.613 ± 0.160 [0.581 0.644] **
	ML + prior	0.828 ± 0.035 [0.821 0.835]( ↑ 30%) **	0.914 ± 0.045 [0.905 0.923] **	0.655 ± 0.074 [0.640 0.670] **
MLP	ML	0.640 ± 0.040 [0.632 0.648]	0.538 ± 0.133 [0.512 0.565]	0.666 ± 0.143 [0.638 0.695]
	PIML	0.843 ± 0.032 [0.837 0.850]( ↑ 32%) **	0.944 ± 0.033 [0.937 0.951] **	0.652 ± 0.066 [0.639 0.666]
	ML + prior	0.833 ± 0.038 [0.825 0.840]( ↑ 30%) **	0.894 ± 0.078 [0.879 0.910] **	0.635 ± 0.094 [0.616 0.654]

*Cervical-G2*				
Model	Prior	0.705 ± 0.001 [0.705 0.705]	0.600 ± 0.451 [0.509 0.688]	0.663 ± 0.254 [0.613 0.714]
DT	ML	0.563 ± 0.051 [0.553 0.573]	0.639 ± 0.122 [0.614 0.663]	0.452 ± 0.166 [0.419 0.485]
	PIML	0.787 ± 0.025 [0.782 0.791]( ↑ 40%) **	0.912 ± 0.016 [0.909 0.916] **	0.559 ± 0.040 [0.551 0.567] **
	ML + prior	0.756 ± 0.020 [0.752 0.760]( ↑ 34%) **	0.943 ± 0.015 [0.940 0.946] **	0.521 ± 0.030 [0.515 0.526] **
RF	ML	0.600 ± 0.013 [0.597 0.602]	0.720 ± 0.101 [0.700 0.740]	0.364 ± 0.180 [0.328 0.399]
	PIML	0.801 ± 0.030 [0.795 0.807]( ↑ 34%) **	0.914 ± 0.027 [0.908 0.919] **	0.538 ± 0.066 [0.525 0.551] **
	ML + prior	0.825 ± 0.009 [0.823 0.827]( ↑ 38%) **	0.906 ± 0.031 [0.900 0.912] **	0.549 ± 0.068 [0.536 0.563] **
SVM	ML	0.583 ± 0.026 [0.577 0.588]	0.757 ± 0.114 [0.735 0.780]	0.345 ± 0.146 [0.316 0.374]
	PIML	0.804 ± 0.025 [0.799 0.809]( ↑ 38%) **	0.893 ± 0.138 [0.865 0.920] **	0.843 ± 0.080 [0.827 0.859] **
	ML + prior	0.799 ± 0.007 [0.797 0.800]( ↑ 37%) **	0.542 ± 0.113 [0.520 0.565] **	0.579 ± 0.092 [0.561 0.597] **
MLP	ML	0.603 ± 0.034 [0.596 0.610]	0.695 ± 0.113 [0.673 0.718]	0.415 ± 0.156 [0.384 0.446]
	PIML	0.801 ± 0.025 [0.796 0.806]( ↑ 33%) **	0.912 ± 0.024 [0.907 0.917] **	0.555 ± 0.045 [0.546 0.565] **
	ML + prior	0.794 ± 0.030 [0.788 0.800]( ↑ 32%) **	0.872 ± 0.061 [0.860 0.884] **	0.561 ± 0.096 [0.542 0.580] **

Results are reported as mean ± SD across 100 repetitions. Prior refers to the prior-only based results. ML + prior refers to direct feature-level prior integration in ML. PIML refers to the proposed Bayesian integration framework with ML. Asterisks indicate statistically significant improvement over baseline ML (Wilcoxon signed-rank test, * p<0.05 ; ** p<0.01 ).

The prior-only method achieved moderate comparable performance, with high specificity but relatively low sensitivity. This indicates that while clinical prior knowledge provides strong rule-based confidence for identifying negative cases, it lacks sufficient flexibility to capture complex patterns required for accurate disease detection. Compared with the prior-only approach, all ML-based methods demonstrated improved sensitivity, indicating the importance of data-driven feature learning in capturing disease-related patterns. Compared with baseline ML, both ML + prior and PIML significantly improved AUC, specificity, and overall diagnostic performance, confirming the effectiveness of incorporating prior knowledge.

Overall, PIML outperformed ML + prior in terms of AUC across most dataset–classifier combinations, with the exception of the RF classifier applied to both Cervical-G1 and Cervical-G2 (p < 0.01; Supplementary Table S4.1; Figure 3). Further evaluation using larger datasets and additional case studies is therefore warranted to identify the conditions and application scenarios under which PIML can consistently outperform alternative approaches. Detailed analyses and extended discussions are provided in the Supplementary Appendix 1.

To further assess the probabilistic interpretation of the PIML output, an external calibration analysis was performed on the Cervical-G2 cohort. The experimental procedure followed the same pipeline as the primary cervical experiment: all model training and parameter estimation were performed using Cervical-G1, while Cervical-G2 was used exclusively as an independent external test cohort. This entire procedure was repeated 100 times using the same repeated experimental design, meaning that the fixed Cervical-G2 cohort was independently evaluated by each of the 100 models trained from the corresponding Cervical-G1 repetitions. Across the 100 repeated experiments, the mean Brier score was 0.116 ± 0.010, and subgroup-based comparisons showed reasonable agreement between the Bayesian-updated probability estimates and observed disease frequencies in several clinical-evidence combinations, although deviations remained in some subgroups because there are only a small number of patients in these subgroups. Detailed calibration results are provided in Supplementary Tables S8, S9 and Supplementary Figure S4.

### 3.2 Task 2: high-risk oral lesion detection

We next applied our method to the oral high-risk lesion dataset to perform binary classification between subjects with high-risk oral lesions and healthy subjects (62 in total) (Kujan et al., 2006). In this task, smoking status was used as prior knowledge. Following the same experimental protocol, we compared four strategies: the prior-only method, baseline machine learning (ML), ML + prior, and PIML. To account for repeated EIS measurements from the same subject, model performance validation and testing were performed at the patient level. Specifically, when multiple EIS readings were available for the same patient, the positive-class prediction scores from these readings were averaged to obtain a single patient-level score. AUC, sensitivity, and specificity were subsequently calculated based on these patient-level scores, so that each patient contributed equally to the performance evaluation irrespective of the number of available EIS readings. The validation threshold was also determined using the corresponding patient-level validation scores. Each experiment was repeated 100 times, and the mean performance metrics with 95% confidence intervals (CI) are reported.

As shown in Table 3, the prior-only method achieved an AUC of 0.827, a specificity of 0.873, and a sensitivity of 0.700, demonstrating the strong association between smoking status and oral high-risk lesion risk. Across the different ML classifiers, the effect of prior-information integration varied according to the underlying classifier. For DT, RF, and MLP, PIML increased the AUC relative to the corresponding ML baseline by approximately 13%, 8%, and 3%, respectively. These improvements were accompanied by marked increases in specificity, from 0.748 to 0.930 for DT, from 0.783 to 0.842 for RF, and from 0.790 to 0.912 for MLP. Sensitivity was also improved for these three classifiers. In contrast, for SVM, direct incorporation of smoking status as an additional ML feature (ML + prior) achieved the highest AUC, whereas PIML produced the highest specificity. Overall, the patient-level results indicate that the effect of prior integration is classifier dependent, while PIML generally improves the specificity of the ML-based diagnosis and provides favorable performance for DT, RF, and MLP. Detailed model-wise comparisons and statistical analyses are provided in Supplementary Appendix 2. To further assess the robustness of the oral-lesion results given the limited number of independent high-risk subjects, we performed an additional sensitivity analysis using 10 repeated stratified nested patient-level 3-fold cross-validation with DT and MLP as representative classifiers. The overall performance patterns were consistent with those observed in the primary analysis, particularly with respect to the improvement in AUC and/or specificity achieved by PIML. These additional results indicate that the main conclusions are not dependent solely on the repeated 8:1:1 hold-out strategy. Detailed results are provided in the Supplementary Material.

**TABLE 3:** Performance comparison on Oral high-risk lesion dataset. * indicate statistically significant improvement over baseline ML (Wilcoxon signed-rank test, *p < 0.05; **p < 0.01).

​	Methods	AUC (95% CI)	Specificity	Sensitivity
Model	Prior	0.827 ± 0.157 [0.806 0.868]	0.873 ± 0.134 [0.847 0.900]	0.700 ± 0.266 [0.647 0.753]
DT	ML	0.726 ± 0.201 [0.687 0.766]	0.748 ± 0.427 [0.664 0.833]	0.535 ± 0.391 [0.457 0.613]
	PIML	0.822 ± 0.165 [0.789 0.855] ( ↑ 13%) **	0.930 ± 0.112 [0.908 0.952] **	0.635 ± 0.347 [0.566 0.704] **
	ML + prior	0.721 ± 0.203 [0.681 0.762]	0.733 ± 0.432 [0.648 0.819]	0.545 ± 0.390 [0.468 0.622]
RF	ML	0.785 ± 0.198 [0.746 0.824]	0.783 ± 0.321 [0.720 0.847]	0.555 ± 0.408 [0.474 0.636]
	PIML	0.848 ± 0.179 [0.812 0.883] ( ↑ 8%) *	0.842 ± 0.248 [0.793 0.891] **	0.607 ± 0.369 [0.548 0.650]
	ML + prior	0.803 ± 0.210 [0.762 0.845]	0.912 ± 0.184 [0.875 0.948] **	0.560 ± 0.378 [0.485 0.635]
SVM	ML	0.756 ± 0.223 [0.712 0.800]	0.718 ± 0.352 [0.648 0.788]	0.540 ± 0.412 [0.458 0.622]
	PIML	0.741 ± 0.198 [0.701 0.780]	0.900 ± 0.169 [0.866 0.933] **	0.547 ± 0.375 [0.472 0.621]
	ML + prior	0.909 ± 0.116 [0.886 0.932] ( ↑ 20%) **	0.882 ± 0.196 [0.843 0.921] **	0.625 ± 0.379 [0.550 0.700]
MLP	ML	0.752 ± 0.230 [0.706 0.797]	0.790 ± 0.280 [0.735 0.846]	0.510 ± 0.389 [0.423 0.577]
	PIML	0.772 ± 0.203 [0.725 0.817] ( ↑ 3%) *	0.912 ± 0.141 [0.884 0.940] **	0.552 ± 0.360 [0.431 0.634]
	ML + prior	0.755 ± 0.234 [0.709 0.802]	0.842 ± 0.272 [0.788 0.896]	0.510 ± 0.369 [0.437 0.583]

The comparative performance is shown in Figure 4 where the ROC curves further illustrate the comparative performance of different methods on the high-risk oral lesion detection task. The ROC curves further illustrate that the performance of PIML and ML + prior varies across classifiers in the high-risk oral lesion detection task. PIML showed improved performance in some classifier settings, whereas ML + prior achieved comparable or higher AUC in others, particularly with the SVM classifier. Nevertheless, both prior-informed strategies demonstrated the potential benefit of incorporating clinically relevant prior information compared with ML-only diagnosis. In particular, the PIML curves tend to reach higher true-positive rates at relatively lower false-positive rates in several classifier settings, suggesting improved diagnostic discrimination. These visual results are consistent with the quantitative findings in Table 3 and further support the robustness and clinical interpretability of the proposed PIML framework.

### 3.3 Task 3: preterm birth diagnosis

We next evaluated model performance on the preterm birth (PTB) dataset, aiming to distinguish between subjects who delivered prematurely and full-term controls. In this task, prior knowledge was derived from each subject’s obstetric history, specifically whether they had previously experienced preterm delivery, a clinically relevant prognostic factor for PTB. Consistent with the previous experiments, we evaluated the prior-only method, baseline ML, ML + prior, and PIML. In addition, we introduced PIML (APP), in which the prior probability was obtained from the publicly available QUiPP tool rather than being estimated from the training data (Carter et al., 2020; Watson et al., 2020). This setting was used to assess the feasibility of incorporating externally derived clinical prior information into the proposed PIML framework.

To account for repeated EIS measurements from the same subject, model evaluation was performed at the patient level. Specifically, when multiple EIS readings were available for the same patient, the positive-class prediction scores from these readings were averaged to obtain a single patient-level score. AUC, sensitivity, and specificity were subsequently calculated based on these patient-level scores, ensuring that each patient contributed equally to the evaluation regardless of the number of EIS readings available. The validation threshold was also determined using the corresponding patient-level validation scores. The results are summarized in Table 4, with detailed model-wise comparisons and statistical analyses provided in Supplementary Appendix 3.

**TABLE 4:** Performance comparison on PTB dataset. * indicate statistically significant improvement over baseline ML (Wilcoxon signed-rank test, *p < 0.05; **p < 0.01).

​	Methods	AUC (95% CI)	Specificity (95% CI)	Sensitivity (95% CI)
Model	Prior	0.685 ± 0.094 [0.667 0.724]	0.608 ± 0.214 [0.566 0.651]	0.757 ± 0.184 [0.721 0.794]
DT	ML	0.498 ± 0.013 [0.496 0.501]	0.005 ± 0.037 [0.002 0.012]	0.993 ± 0.051 [0.983 1.000]
	PIML	0.704 ± 0.095 [0.685 0.723] ( ↑ 41%) **	0.610 ± 0.215 [0.567 0.652] **	0.754 ± 0.185 [0.718 0.791] **
	ML + prior	0.605 ± 0.111 [0.583 0.627] ( ↑ 21%) **	0.361 ± 0.354 [0.291 0.432] **	0.796 ± 0.252 [0.746 0.846] **
	PIML (APP)	0.692 ± 0.100 [0.675 0.711] ( ↑ 39%) **	0.600 ± 0.114 [0.562 0.631] **	0.745 ± 0.105 [0.723 0.781] **
RF	ML	0.448 ± 0.116 [0.425 0.471]	0.358 ± 0.249 [0.308 0.407]	0.576 ± 0.275 [0.521 0.630]
	PIML	0.693 ± 0.111 [0.661 0.705] ( ↑ 52%) **	0.649 ± 0.161 [0.617 0.681] **	0.679 ± 0.238 [0.631 0.726] **
	ML + prior	0.657 ± 0.106 [0.636 0.678] ( ↑ 47%) **	0.600 ± 0.165 [0.567 0.633] **	0.663 ± 0.218 [0.620 0.706] **
	PIML (APP)	0.676 ± 0.117 [0.652 0.699] ( ↑ 51%) **	0.648 ± 0.172 [0.614 0.683] **	0.664 ± 0.245 [0.616 0.713] **
SVM	ML	0.465 ± 0.087 [0.442 0.488]	0.513 ± 0.291 [0.456 0.571]	0.454 ± 0.338 [0.387 0.521]
	PIML	0.666 ± 0.119 [0.643 0.690] ( ↑ 43%) **	0.539 ± 0.298 [0.480 0.598]	0.558 ± 0.271 [0.504 0.611] **
	ML + prior	0.460 ± 0.146 [0.431 0.488]	0.593 ± 0.317 [0.530 0.656] *	0.370 ± 0.322 [0.306 0.434] *
	PIML (APP)	0.653 ± 0.121 [0.629 0.678] ( ↑ 40%) **	0.537 ± 0.296 [0.478 0.595]	0.549 ± 0.271 [0.495 0.603] **
MLP	ML	0.488 ± 0.117 [0.465 0.511]	0.394 ± 0.238 [0.347 0.442]	0.594 ± 0.286 [0.538 0.651]
	PIML	0.680 ± 0.103 [0.649 0.691] ( ↑ 37%) **	0.668 ± 0.164 [0.635 0.700] **	0.650 ± 0.235 [0.603 0.697]
	ML + prior	0.651 ± 0.105 [0.630 0.671] ( ↑ 33%) **	0.613 ± 0.210 [0.571 0.654] **	0.567 ± 0.269 [0.514 0.621]
	PIML (APP)	0.664 ± 0.107 [0.643 0.685] ( ↑ 36%) **	0.670 ± 0.155 [0.639 0.701] **	0.644 ± 0.232 [0.598 0.690] **

We additionally verified that PTB was consistently defined as the positive class for all classifiers and that the ROC score used for AUC calculation always increased with predicted PTB risk. Therefore, the near-chance or below-chance AUC values were not attributable to label inversion or score-direction errors, but reflected the limited discriminative capability of the baseline models under the unified, non-PTB-specific feature-processing pipeline.

As shown in Table 4, PIML substantially improved the discriminative performance of the baseline ML models across all four classifiers. Compared with baseline ML, PIML increased AUC by approximately 41%, 52%, 43%, and 37% for DT, RF, SVM, and MLP, respectively. These improvements were accompanied by marked increases in specificity and, in most cases, improved sensitivity. The effect was particularly evident for DT, where baseline ML showed extremely high sensitivity but almost no specificity, whereas PIML produced a substantially more balanced sensitivity-specificity profile.

PIML (APP) showed a similar overall pattern. Its AUC values were 0.692, 0.676, 0.653, and 0.664 for DT, RF, SVM, and MLP, respectively, and were consistently close to those obtained using the internally estimated priors. This demonstrates that the PIML framework is not restricted to prior probabilities estimated from the training cohort and can also accommodate externally derived clinical risk information.

The prior-only method also showed substantial predictive capability, achieving an AUC of 0.685, specificity of 0.608, and sensitivity of 0.757. This indicates that previous PTB history itself contains clinically meaningful prognostic information. Nevertheless, the PIML framework provides a systematic mechanism for combining such prior clinical information with EIS-derived ML evidence, thereby improving otherwise weak or poorly balanced ML classifiers and enabling more reliable patient-specific diagnostic decision-making. As illustrated in Figure 5, both PIML and PIML (APP) demonstrate consistently superior ROC performance across all classifiers, characterized by a noticeable improvement. Notably, the ROC curves of ML and ML + prior methods reveal strong variability across models and generally lower performance in the mid-to-high specificity range.

## 4 Discussion

This study introduces a prior-informed machine learning (PIML) framework that enables structured prior knowledge to inform and enhance ML–based diagnostic decisions. Across three independent diagnostic tasks, cervical neoplasia detection, high-risk oral lesion detection, and preterm birth detection, PIML generally improved performance relative to the corresponding baseline ML models, and outperformed existing prior-only and feature-level prior integration strategies in most dataset–classifier combinations. These findings reveal that prior domain knowledge, when integrated into the probabilistic inference process rather than treated merely as additional input features, can potentially enhance both the accuracy and interpretability of machine learning–based medical diagnosis. In the context of PIML, “clinical interpretability” refers specifically to the transparency and traceability of the diagnostic updating process rather than to an explanation of the internal feature representations learned by the underlying ML classifier. Instead of providing only a binary diagnostic output, PIML explicitly shows how a patient-specific clinical prior is updated by ML-derived diagnostic evidence, taking into account the empirically determined sensitivity and specificity of the ML model, to produce a posterior probability of disease. The resulting posterior probability therefore has a direct probabilistic interpretation and allows clinicians to understand how the initial clinical risk and the additional ML evidence jointly contribute to the final diagnostic outcome.

For AI-based medical diagnosis, explainability and interpretability are essential for building clinical trust, ensuring accountability, identifying potential biases, and promoting the responsible use of AI in healthcare (Tan and Benos, 2025). The explainability and interpretability offered by PIML can make significant contributions towards achieving these objectives. PIML provides a clearer understanding of how diagnostic decisions are derived by combining ML-based predictions with inferences based on medical knowledge. The sensitivity and specificity of the ML model provide valuable information about its diagnostic performance and help identify potential sources of bias in its predictions. Meanwhile, the medical knowledge-based prior represents the diagnostic insight that can be obtained from existing clinical knowledge and expertise. Bayesian inference not only provides a coherent framework for integrating ML predictions with medical knowledge but also offers a rigorous mechanism for tracing the contributions of these components to the final diagnostic outcome. This transparency supports accountability and, when combined with appropriate governance and human oversight, facilitates the fair, privacy-preserving, and responsible integration of ML into clinical workflows. In addition, given the broad range of expertise required, the development of PIML for specific medical applications should involve cross-disciplinary collaboration, with clinical experts playing a critical role in ensuring that the resulting systems are clinically relevant, appropriately contextualized, and aligned with real-world healthcare needs. A patient-level illustrative example of this clinical interpretable process is provided in the Supplementary Material (see Supplementary case).

Notably, in Task 1 (cervical neoplasia detection) and Task 2 (high-risk oral lesion detection), PIML achieved better diagnostic performance than the ML + prior approach in most cases, with comparable performance in a few instances. In both tasks, the prior probabilities (P(H|C)), were derived directly from the distributions of the internal training data. The comparable performance observed in the few instances may be attributed to the fact that, despite employing different computational strategies, both ML + prior and PIML draw on the same knowledge source: structured priors estimated from the same population.

In Task 3, the proposed PIML framework achieved a substantial improvement over all other methods. The weak baseline ML performance observed in the PTB task should be interpreted in the context of the characteristics of this dataset. Multiple EIS readings are available for individual subjects and may vary in measurement quality and predictive relevance. Previous work by Tian et al. (2023) introduced a dedicated filter-predictor strategy together with additional feature construction for this PTB-EIS setting, highlighting the difficulty of modelling these measurements using a conventional prediction pipeline. In the present study, we intentionally did not introduce PTB-specific reading selection or complex feature engineering, because the objective was to evaluate PIML using the same general ML pipeline across all three diagnostic applications. The baseline models therefore used the Cole-model-derived EIS features without additional PTB-specific optimization. The near-chance baseline performance should consequently be interpreted as limited discrimination of EIS-only conventional ML under this experimental setting, rather than as the performance of an optimized PTB-specific prediction model.

In addition, when the ML model performs poorly, as observed in Task 3, PIML generally improves upon the ML + prior method, a conventional approach for incorporating auxiliary information to improve model performance. This finding suggests that PIML can potentially provide a significantly improved alternative to conventional feature-level prior fusion in settings where baseline ML performance is limited, although broader comparative studies with other fusion strategies are required to further confirm the conclusion.

Another important observation is that when both the ML model and prior knowledge exhibit limited predictive performance individually, the proposed PIML yields the best overall results. This phenomenon is evident in both Task 1 and Task 3. In contrast, Task 2 does not show the same, as both ML and prior based methods already achieve strong performance (AUC>0.80). This may be attributed to the relatively small oral high-risk lesion dataset size and the weaker predictive power of smoking status in Task 2 compared to key risk factors such as PTB history or cytology results in the other two tasks. However, the high-risk oral lesion detection results should therefore be interpreted with particular caution because this cohort comprised only 62 subjects, including 9 high-risk cases. Such clinically verified EIS datasets are difficult to acquire because their collection requires prospective patient recruitment, device-based measurements, and corresponding clinical or histopathological diagnostic information. Although stratified subject-level splitting ensured representation of high-risk cases in the training, validation, and test subsets, and repeated evaluation across 100 predefined random seeds reduced sensitivity to any particular data partition, these repetitions do not increase the number of independent patients. The limited number of high-risk subjects therefore remains an important source of uncertainty, particularly for sensitivity estimation. Accordingly, the high-risk oral lesion detection experiment should be regarded as a proof-of-concept evaluation of the applicability of PIML, and further validation using a larger independent cohort will be required to establish its clinical generalizability.

Methodologically, the superior performance of PIML across all three tasks can be attributed to its two core conceptual contributions. First, it effectively integrates ML–derived diagnostic predictions with structured clinical priors, moving beyond the simple feature-level fusion approaches commonly adopted in ML applications. Second, and perhaps most importantly, this study embeds the determination of empirical sensitivity and specificity required for Bayesian inference directly within the end-to-end ML model-building process. In contrast, traditional Bayesian diagnostic approaches rely on predefined or *a priori* known distributions (Chatzimichail and Hatjimihail, 2024), an assumption that is often difficult to satisfy in real-world clinical settings where the underlying distribution is unknown. The PIML framework integrates a complete ML pipeline, including model training, validation, testing, and optimal threshold determination, with downstream Bayesian updating. By optimizing classification thresholds within this unified framework, PIML not only enhances the baseline diagnostic performance of the ML model but also systematically enables the derivation of clinically meaningful and Bayesian-updated posterior diagnostic estimates.

In real-world settings, prior knowledge may be drawn from heterogeneous sources, including population-level screening data, hospital-specific statistics, commercial tools (e.g., QUiPP), and domain expertise accumulated through clinical practice. Unlike traditional machine learning models that require retraining to incorporate new input features, the proposed PIML framework enables *post hoc* adaptation at inference by updating the outputs of a trained model using revised prior probabilities P(H|C). This capacity to recontextualize predictions considering evolving prior knowledge makes PIML particularly well suited to dynamic clinical environments, where disease prevalence, diagnostic practices, and population characteristics may vary across time and location. Although the present study was validated using EIS-derived features, our PIML framework is not restricted to EIS: the underlying ML model can process features derived from other data modalities, including medical imaging, laboratory biomarkers or different combinations of modalities. Future work should therefore evaluate the framework using multimodal datasets and prospective, multi-center cohorts to establish its generalizability across different diseases, populations and clinical workflows.

Compared with data-driven fusion approaches such as logistic regression-based late fusion and probability stacking, PIML is methodologically distinct in that it explicitly separates patient-specific clinical prior information from ML-derived diagnostic evidence and integrates them coherently through Bayesian updating. In many existing data-driven fusion strategies, an additional model is learned from a specific development cohort to determine how ML outputs and clinical variables are combined. In contrast, the clinical prior in PIML is not required to originate from the same training dataset used to develop the ML model and may instead be derived from external clinical tools, population-level epidemiological statistics, or established clinical knowledge. These differences highlight the conceptual and methodological advantages of the PIML framework, although these advantages were not empirically demonstrated in the present study, as the clinical priors were derived from the training data in most cases due to the lack of additional independent information for their estimation.

The findings in the present study should be interpreted in relation to the level of validation achieved in each application. Evaluation across cervical neoplasia detection, high-risk oral lesion detection, and preterm birth prediction demonstrates the applicability of PIML across different diagnostic tasks and prior-information settings, but does not by itself establish broad clinical generalizability. Among the three applications, only Cervical-G2 provides evaluation in an independent external cohort. The oral and PTB analyses rely on repeated internal resampling of their respective source cohorts and therefore represent internal evaluation. In addition, the PIML (APP) experiment demonstrates the feasibility of incorporating an externally developed clinical prior but does not constitute an external validation of the underlying diagnostic model. Accordingly, broader generalizability and translational utility remain to be established through prospective, multicenter validation in larger independent cohorts.

The current implementation of the PIML framework assumes conditional independence between the ML-derived evidence and the clinical prior information given the disease status. Although this assumption may be clinically plausible in many cases, given the complementary nature of the ML-derived evidence and clinical prior information, larger datasets are needed to empirically assess its validity. Where this assumption is substantially violated, incorporating clinical-information-specific likelihoods or joint probabilistic models may be necessary when applying the PIML framework. Investigating these extensions and empirically evaluating the conditional independence assumption will therefore be important priorities for our future work. Another limitation is that the sample size of oral EIS dataset in this study is limited. The oral-lesion experiment should be interpreted with particular caution because the cohort comprised only 62 subjects, including nine high-risk cases. Although all data partitions were performed at the subject level using a stratified procedure to preserve representation of both classes and prevent information leakage, the small number of independent high-risk subjects inevitably limits the stability of performance estimates, particularly sensitivity, AUC and validation-derived threshold selection. The 100 repeated partitions were therefore used to assess the robustness of the observed results to different patient-level data partitions, rather than to represent 100 independent clinical samples or to increase the effective sample size. Because individual subjects can appear in different partitions across repetitions, the resulting statistical comparisons and uncertainty estimates should be interpreted as reflecting the consistency of method-wise differences across repeated partitions rather than the precision that would be obtained from 100 independent cohorts. Accordingly, the oral experiment should be regarded as a proof-of-concept evaluation of the applicability of PIML, and validation in a substantially larger independent cohort will be required to establish its clinical generalizability. In the present analysis, repeated EIS measurements from the same patient were aggregated at the prediction-score level for validation and test evaluation, ensuring that each patient contributed only one observation to the reported performance metrics. Future studies with larger cohorts may further investigate alternative approaches that explicitly model within-subject measurement variability.

Although PIML generates probability-valued outputs through Bayesian updating, these outputs should be interpreted as Bayesian-updated probability estimates under the stated assumptions. In the present study, we performed an initial external calibration assessment using the independent Cervical-G2 cohort, with DT selected as a representative classifier. Across 100 repeated experiments, the mean Brier score was 0.116 ± 0.010, with a range of 0.096–0.136. In addition, the PIML probability estimates were compared with the corresponding observed disease rates across eight combinations of ML diagnostic evidence, smear-test result, and colposcopy result. Several subgroups showed relatively good agreement, although noticeable deviations remained for some other subgroup combinations, likely due to the small number of patients in these subgroups. Therefore, more comprehensive calibration analyses using larger independent cohorts, additional classifiers, and complementary measures, such as the calibration intercept and slope and decision-curve analysis, will be important areas for further research.

## Data Availability

The original contributions presented in the study are included in the article/Supplementary Material, further inquiries can be directed to the corresponding author.

## References

[B1] Ahuja A. S. (2019). The impact of artificial intelligence in medicine on the future role of the physician. PeerJ 7, e7702. 10.7717/peerj.7702 31592346 PMC6779111

[B2] Ananda Rao A. Awale M. Davis S. (2023). Medical diagnosis reimagined as a process of Bayesian reasoning and elimination. Cureus 15, e45097. 10.7759/cureus.45097 37705565 PMC10497324

[B3] Baaj I. Bouraoui Z. Cornuéjols A. Denœux T. Destercke S. Dubois D. (2024). Synergies between machine learning and reasoning - an introduction by the kay R. Amel group. Int. J. Approx. Reason. 171, 109206. 10.1016/j.ijar.2024.109206

[B4] Baker A. Perov Y. Middleton K. Baxter J. Mullarkey D. Sangar D. (2020). A comparison of artificial intelligence and human doctors for the purpose of triage and diagnosis. Front. Artif. Intell. 3, 543405. 10.3389/frai.2020.543405 33733203 PMC7861270

[B5] Bonnasse-Gahot L. (2022). Interpolation, extrapolation, and local generalization in common neural networks. arXiv arXiv:2207.08648. 10.48550/arXiv.2207.08648

[B6] Brown B. H. Tidy J. A. Boston K. Blackett A. D. Smallwood R. H. Sharp F. (2000). Relation between tissue structure and imposed electrical current flow in cervical neoplasia. Lancet 355 (9207), 892–895. 10.1016/S0140-6736(99)09095-9 10752706

[B7] Brown B. H. Milnes P. Abdul S. Tidy J. A. (2005). Detection of cervical intraepithelial neoplasia using impedance spectroscopy: a prospective study. BJOG Int. J. Obstet. Gynaecol. 112 (6), 802–806. 10.1111/j.1471-0528.2004.00530.x 15924541

[B8] Buendia R. Gil-Pita R. Seoane F. (2011). “Cole parameter estimation from total right side electrical bioimpedance spectroscopy measurements — influence of the number of frequencies and the upper limit,” in 2011 Annual International Conference of the IEEE Engineering in Medicine and Biology Society (Boston, MA: IEEE), 1843–1846. 10.1109/IEMBS.2011.6090524 22254688

[B9] Carter J. Seed P. T. Watson H. A. David A. L. Sandall J. Shennan A. H. (2020). Development and validation of predictive models for QUiPP App v.2: tool for predicting preterm birth in women with symptoms of threatened preterm labor. Ultrasound Obstet. Gynecol. 55 (3), 357–367. 10.1002/uog.20422 31385343

[B10] Chatzimichail T. Hatjimihail A. T. (2024). A software tool for applying Bayes’ theorem in medical diagnostics. BMC Med. Inf. Decis. Mak. 24 (1), 399. 10.1186/s12911-024-02721-x 39709395 PMC11662465

[B11] Choi E. Bahadori M. T. Schuetz A. Stewart W. F. Sun J. (2016). Doctor AI: predicting clinical events *via* recurrent neural networks. arXiv arXiv:1511.05942. 10.48550/arXiv.1511.05942 PMC5341604 28286600

[B12] Donnat C. Miolane N. Bunbury F. Kreindler J. (2026). A Bayesian Hierarchical Network for Combining Heterogeneous Data Sources in Medical Diagnoses.

[B13] Duta I. Kariuki S. M. Ngugi A. K. Mwesige A. K. Masanja H. Mwanga D. M. (2025). Evaluating the generalisability of region-naïve machine learning algorithms for the identification of epilepsy in low-resource settings. PLOS Digit. Health 4 (2), e0000491. 10.1371/journal.pdig.0000491 39937713 PMC11819582

[B14] Fang C. Arango Argoty G. A. Kagiampakis I. Khalid M. H. Jacob E. Bulusu K. C. (2024). Integrating knowledge graphs into machine learning models for survival prediction and biomarker discovery in patients with non–small-cell lung cancer. J. Transl. Med. 22 (1), 726. 10.1186/s12967-024-05509-9 39103897 PMC11302075

[B15] Göndöcs D. Dörfler V. (2024). AI in medical diagnosis: AI prediction & human judgment. Artif. Intell. Med. 149, 102769. 10.1016/j.artmed.2024.102769 38462271

[B16] Hinton G. (2024). Hinton‘S Presentation at the Royal Swedish Academy of Engineering Sciences (IVA) in Stockholm on 5 December 2024. Available online at: https://www.youtube.com/watch?v=lexF-CrhOrE (Accessed October 19, 2026).

[B17] Hughes C. A. Gose E. E. Roseman D. L. (1990). Overcoming deficiencies of the rule-based medical expert system. Comput. Methods Programs Biomed. 32, 63–72. 10.1016/0169-2607(90)90086-o 2401135

[B18] Jiang J. Wang H. Xie J. Guo X. Guan Y. Yu Q. (2020). Medical knowledge embedding based on recursive neural network for multi-disease diagnosis. Artif. Intell. Med. 103, 101772. 10.1016/j.artmed.2019.101772 32143787

[B19] Jospin L. V. Buntine W. Boussaid F. Laga H. Bennamoun M. (2022). Hands-on bayesian neural networks -- a tutorial for deep learning users. IEEE Comput. Intell. Mag. 17 (2), 29–48. 10.1109/MCI.2022.3155327

[B20] Juravle G. Boudouraki A. Terziyska M. Rezlescu C. (2020). Chapter 14 - trust in artificial intelligence for medical diagnoses. Prog. Brain Res. 253, 263–282. 10.1016/bs.pbr.2020.06.006 32771128

[B21] Kelly C. J. Karthikesalingam A. Suleyman M. Corrado G. King D. (2019). Key challenges for delivering clinical impact with artificial intelligence. BMC Med. 17 (1), 195. 10.1186/s12916-019-1426-2 31665002 PMC6821018

[B22] Khan S. Z. Farooq A. Masood M. Shahid A. Khan I. U. Nisar H. (2020). Smokeless tobacco use and risk of oral cavity cancer. Turk. J. Med. Sci. 44 (2), 291–297. 10.3906/sag-1809-11 31655504 PMC7165244

[B23] Kujan O. Oliver R. J. Khattab A. Roberts S. A. Thakker N. Sloan P. (2006). Evaluation of a new binary system of grading oral epithelial dysplasia for prediction of malignant transformation. Oral Oncol. 42 (10), 987–993. 10.1016/j.oraloncology.2005.12.014 16731030

[B24] Kunhimangalam R. Ovallath S. Joseph P. K. (2014). A clinical decision support system with an integrated EMR for diagnosis of peripheral neuropathy. J. Med. Syst. 38 (4), 38. 10.1007/s10916-014-0038-9 24692180

[B25] Kyrimi E. Neves M. R. McLachlan S. Neil M. Marsh W. Fenton N. (2020). Medical idioms for clinical Bayesian network development. J. Biomed. Inf. 108, 103495. 10.1016/j.jbi.2020.103495 32619692

[B26] Lakhani P. Sundaram B. (2017). Deep learning at chest radiography: automated classification of pulmonary tuberculosis by using convolutional neural networks. Radiology 284 (2), 574–582. 10.1148/radiol.2017162326 28436741

[B27] Leiser F. Rank S. Schmidt-Kraepelin M. Thiebes S. Sunyaev A. (2023). Medical informed machine learning: a scoping review and future research directions. Artif. Intell. Med. 145, 102676. 10.1016/j.artmed.2023.102676 37925206

[B28] Leite D. Andrade E. Rativa D. Maciel A. M. A. (2024). Fault detection and diagnosis in industry 4.0: a review on challenges and opportunities. Sensors 25 (1), 60. 10.3390/s25010060 39796851 PMC11723332

[B29] Li P. Highfield P. E. Lang Z.-Q. Kell D. (2021). Cervical cancer prognosis and diagnosis using electrical impedance spectroscopy. J. Electr. Bioimpedance 12 (1), 153, 162. 10.2478/joeb-2021-0018 35069951 PMC8713385

[B30] Li Q. Ren Z. Cao K. Li M. M. Wang K. Zhou Y. (2022). CancerVar: an artificial intelligence–empowered platform for clinical interpretation of somatic mutations in cancer. Sci. Adv. 10.1126/sciadv.abj1624 PMC9075800 35544644

[B31] Li X. He Y. Wu J. Wang Z. Zhao X. Zhang Y. (2026). PathTIGR: a pathway topology-informed graph representation learning framework for immunotherapy response prediction. Sci. Adv. 12 (24), eaed6373. 10.1126/sciadv.aed6373 42268974 PMC13251857

[B32] Lin Z. Lang Z.-Q. Guo L. Walker D. C. Wang M. Murdoch C. (2026). 1 Deep Learning-based Electrical Impedance Spectroscopy Analysis for 2 Malignant and Potentially Malignant Oral Disorder Detection. 10.1038/s41598-025-05116-8 PMC12134277 40461631

[B33] Lin Z. Henson W. H. Dowling L. Walsh J. Dall’Ara E. Guo L. (2024). Automatic segmentation of skeletal muscles from MR images using modified U-Net and a novel data augmentation approach. Front. Bioeng. Biotechnol. 12, 1355735. 10.3389/fbioe.2024.1355735 38456001 PMC10919285

[B34] Liu X. Faes L. Kale A. U. Wagner S. K. Fu D. J. Bruynseels A. (2019). A comparison of deep learning performance against health-care professionals in detecting diseases from medical imaging: a systematic review and meta-analysis’, Lancet Digit. Health, 1 (6), e271–e297. 10.1016/S2589-7500(19)30123-2 33323251

[B36] Muehlemann N. Zhou T. Mukherjee R. Hossain M. I. Roychoudhury S. Russek-Cohen E. (2023). A tutorial on modern bayesian methods in clinical trials. Ther. Innov. Regul. Sci. 57 (3), 402–416. 10.1007/s43441-023-00515-3 37081374 PMC10117244

[B37] Murdoch C. Brown B. H. Hearnden V. Speight P. M. D'Apice K. Hegarty A. M. (2014). Use of electrical impedance spectroscopy to detect malignant and potentially malignant oral lesions. Int. J. Nanomedicine 9, 4521–4532. 10.2147/IJN.S64087 25285005 PMC4181751

[B38] Narice B. F. Labib M. Wang M. Byrne V. Shepherd J. Lang Z. Q. (2024). Developing a logistic regression model to predict spontaneous preterm birth from maternal socio-demographic and obstetric history at initial pregnancy registration. BMC Pregnancy Childbirth 24 (1), 688. 10.1186/s12884-024-06892-3 39433994 PMC11494931

[B39] Nishtar T. Burki S. Ahmad F. S. Ahmad T. (2021). Diagnostic accuracy of computer aided reading of chest x-ray in screening for pulmonary tuberculosis in comparison with gene-Xpert. Pak. J. Med. Sci. 38 (1). 10.12669/pjms.38.1.4531 35035402 PMC8713241

[B40] Orouji S. Liu M. C. Korem T. Peters M. A. K. (2024). Domain adaptation in small-scale and heterogeneous biological datasets. Sci. Adv. 10 (51), eadp6040. 10.1126/sciadv.adp6040 39705361 PMC11661433

[B41] Quinn T. P. Senadeera M. Jacobs S. Coghlan S. Le V. (2021). Trust and medical AI: the challenges we face and the expertise needed to overcome them. J. Am. Med. Inf. Assoc. 28 (4), 890–894. 10.1093/jamia/ocaa268 33340404 PMC7973477

[B35] Rothman J. (2026). Why the godfather of A.I. fears what he’s built. Available online at: https://www.newyorker.com/magazine/2023/11/20/geoffrey-hinton-profile-ai (Accessed October 19, 2026).

[B42] Sheikhtaheri A. Sadoughi F. Hashemi Dehaghi Z. (2014). Developing and using expert systems and neural networks in medicine: a review on benefits and challenges. J. Med. Syst. 38 (9), 110. 10.1007/s10916-014-0110-5 25027017

[B43] Shen J. Zhang C. J. P. Jiang B. Chen J. Song J. Liu Z. (2019). Artificial intelligence *versus* clinicians in disease diagnosis: systematic review. JMIR Med. Inf. 7 (3), e10010. 10.2196/10010 31420959 PMC6716335

[B44] Sirocchi C. Bogliolo A. Montagna S. (2024). Medical-informed machine learning: integrating prior knowledge into medical decision systems. BMC Med. Inf. Decis. Mak. 24 (S4), 186. 10.1186/s12911-024-02582-4 38943085 PMC11212227

[B45] Stewart R. Ermon S. (2017). Label-free supervision of neural networks with physics and domain knowledge. Proc. AAAI Conf. Artif. Intell. 31 (1). 10.1609/aaai.v31i1.10934

[B46] Tan M. J. T. Benos P. V. (2025). Addressing intersectionality, explainability, and ethics in AI-Driven diagnostics: a rebuttal and call for transdiciplinary action. arXiv arXiv:2501.08497. 10.48550/arXiv.2501.08497

[B47] Tian D. Lang Z.-Q. Zhang D. Anumba D. O. (2023). A filter-predictor polynomial feature based machine learning approach to predicting preterm birth from cervical electrical impedance spectroscopy. Biomed. Signal Process. Control 80, 104345. 10.1016/j.bspc.2022.104345

[B48] Tidy J. Brown B. H. Healey T. J. Daayana S. Martin M. Prendiville W. (2013). Accuracy of detection of high‐grade cervical intraepithelial neoplasia using electrical impedance spectroscopy with colposcopy. BJOG Int. J. Obstet. Gynaecol. 120 (4), 400–411. 10.1111/1471-0528.12096 23289897 PMC3597993

[B49] Topol E. J. (2019). High-performance medicine: the convergence of human and artificial intelligence. Nat. Med. 25 (1), 44–56. 10.1038/s41591-018-0300-7 30617339

[B50] Tu T. Schaekermann M. Palepu A. Saab K. Freyberg J. Tanno R. (2025). Towards conversational diagnostic artificial intelligence. Nature 642 (8067), 442–450. 10.1038/s41586-025-08866-7 40205050 PMC12158756

[B51] von Rueden L. Mayer S. Beckh K. Georgiev B. Giesselbach S. Heese R. (2021). Informed machine learning -- A taxonomy and survey of integrating knowledge into learning systems. IEEE Trans. Knowl. Data Eng. 35 (1), 614–633. 10.1109/TKDE.2021.3079836

[B52] Wang D. Khosla A. Gargeya R. Irshad H. Beck A. H. (2016). Deep learning for identifying metastatic breast cancer. arXiv arXiv:1606.05718. 10.48550/arXiv.1606.05718

[B53] Wang Y. King R. D. (2023). “Extrapolation is not the same as interpolation”, in *Discovery Science*, Vol. 14276. Bifet A. Lorena A. C. Ribeiro R. P. Gama J. Abreu P. H. , Eds 14276 Cham: Springer Nature Switzerland, pp. 277–292. 10.1007/978-3-031-45275-8_19

[B54] Watson H. A. Seed P. T. Carter J. Hezelgrave N. L. Kuhrt K. Tribe R. M. (2020). Development and validation of predictive models for QUiPP app v.2: tool for predicting preterm birth in asymptomatic high‐risk women. Ultrasound Obstet. Gynecol. 55 (3), 348–356. 10.1002/uog.20401 31325332

[B55] Yang Y. Liu Y. Liu X. Gulhane A. Mastrodicasa D. Wu W. (2025). Demographic bias of expert-level vision-language foundation models in medical imaging. Sci. Adv. 11 (13), eadq0305. 10.1126/sciadv.adq0305 40138420 PMC11939055

[B56] Yang J. Soltan A. A. S. Clifton D. A. (2022). Machine learning generalizability across healthcare settings: insights from multi-site COVID-19 screening. Npj Digit. Med. 5 (1), 69. 10.1038/s41746-022-00614-9 35672368 PMC9174159

[B57] Zech J. R. Badgeley M. A. Liu M. Costa A. B. Titano J. J. Oermann E. K. (2018). Variable generalization performance of a deep learning model to detect pneumonia in chest radiographs: a cross-sectional study. PLOS Med. 15 (11), e1002683. 10.1371/journal.pmed.1002683 30399157 PMC6219764

