# Supplementary material for: Prior-informed machine learning for clinically interpretable medical diagnosis
Source: Front Bioeng Biotechnol. 2026 Oct 28;14:1973664. doi: 10.3389/fbioe.2026.1973664 (PMC13750749; doi:10.3389/fbioe.2026.1973664)
Supplement: Supplementary file 1 [file Supplementaryfile1.docx]

Supplementary Material

# 1 Supplementary Figures and Tables

## 1.1 Supplementary Figures

**Supplementary Figure 1.** **External prior probability estimation using QUiPP App.** (a) Screenshot of the QUiPP input interface, where user-entered clinical parameters (e.g., previous preterm delivery, cervical length, fFN levels) are used to compute risk estimates. (b) Output showing 13.7% risk of spontaneous delivery before 37 weeks when no prior preterm birth is recorded. (c) Output increases to 37.4% under identical input conditions except for selecting “Yes” to prior preterm delivery.

To obtain an external estimate of the prior probability $P(H)$ for preterm birth, we utilized the publicly available QUiPP App, a clinically validated tool designed for assessing individualized risk of spontaneous delivery. This app integrates clinical parameters—such as cervical length, fetal fibronectin (fFN) levels, and history of preterm delivery—to compute a personalized probability of delivery before key gestational thresholds (e.g., 30, 34, and 37 weeks).

In actual clinical settings, practitioners can enter patient-specific information into the QUiPP interface (see Supplementary Figure 1a), and the app generates a visual risk estimation (Supplementary Figure 1b,c). In our implementation, we focused on the predicted probability of delivery before 37 weeks, and this value was used as the prior probability $P(H)$ in our PIML(APP) framework.

However, due to limitations in the available dataset, not all input variables required by QUiPP were recorded for every subject. To maintain consistency across all samples, we fixed the input values for all features except the history of previous preterm birth (see Supplementary Figure 1a), which was available for every subject and entered accordingly (either “Yes” or “No”). The resulting differences in predicted risk are illustrated: for example, with otherwise identical inputs, the probability of preterm birth rises from 13.7% in the absence of prior history to 37.4% when previous preterm delivery is present.

**Supplementary Figure 2. Detailed workflow for ML model training, threshold tuning, and Bayesian evaluation in PIML.**

This flowchart illustrates the complete experimental workflow adopted for all three tasks in our study. The dataset was randomly partitioned into training (80%), validation (10%), and testing (10%) sets. The procedure was repeated 100 times with different random seeds (MATLAB 2022a) to ensure statistical robustness and repeatability.

On the left branch, hyperparameter tuning was performed by training machine learning (ML) models—including decision tree (DT), random forest (RF), support vector machine (SVM), and multilayer perceptron (MLP)—on the training set and validating their performance (AUC) on the validation set to select the optimal configuration.

On the right branch, after ML model architecture was fixed, the test evaluation phase began. The ML model was retrained on the training set and evaluated on both the validation and test sets to obtain output scores. A threshold $\tau_{1}$ was selected based on the ROC curve of the validation set and applied to the test scores to compute original test sensitivity and specificity.

Subsequently, Bayesian inference was conducted. The prior probability $P(H)$ was estimated from either training set statistics or external sources (e.g., QUiPP app in Task 3). Validation sensitivity and specificity values were combined with test scores to compute posterior probabilities via Bayes' inference. A second threshold $\tau_{2}$ was then determined from the validation set under Bayesian adjustment and used to derive final sensitivity and specificity on the test set after Bayesian correction.

This two-stage process ensures that model performance is evaluated in both conventional and Bayesian-integrated (PIML) settings, enabling comparison across ML-only, ML+prior, and PIML strategies.

**Supplementary Figure 3. Comprehensive ROC comparison under different prior knowledge configurations for cervical neoplasia detection.** Receiver operating characteristic (ROC) curves on the Cervical-G1 dataset (a–d) and external validation Cervical-G2 dataset (e–h) using four machine learning models: decision tree (DT), random forest (RF), support vector machine (SVM), and multilayer perceptron (MLP). Each subplot includes multiple configurations: Red solid line: ML baseline without prior knowledge. Blue solid line: PIML integrating both colposcopy and smear test. Green dashed line: ML+prior by direct concatenation of both priors. Yellow solid line: PIML with only smear test. Green solid line: PIML with only colposcopy. Yellow dashed line: ML+prior with only smear test. Blue dashed line: ML+prior with only colposcopy.

The ROC curves shown correspond to a single representative trial (out of 100 repetitions in total), and the red diamond marker indicates the threshold-based working point selected on the validation set. These comparisons highlight the added value of different prior sources as well as the advantage of PIML over original ML and feature-level fusion.

**Supplementary Figure 4. External calibration of PIML Bayesian-updated probability estimates on the Cervical-G2 cohort.** Patients were classified into 8 groups according to the binary ML diagnostic evidence, smear-test result, and colposcopy result. Each point represents one of the eight combinations and compares the mean PIML Bayesian-updated probability estimate across 100 repeated experiments with the corresponding mean observed disease rate in the external cohort. The dashed diagonal line represents perfect agreement between the estimated probability and the observed disease frequency, with points closer to the diagonal indicating better agreement.

## 1.2 Supplementary Tables

Table S1 Performance comparison on Cervical-G1/G2 dataset (prior knowledge used: colposcopy examination). All results are reported as mean value across 100 repetitions. ‘ML+prior’ refers to direct feature-level prior integration in ML. ‘PIML’ refers to our proposed Bayesian integration framework with ML. Asterisks indicate statistically significant improvement over baseline ML (Wilcoxon signed-rank test, * :$\boldsymbol{p<0.05}$; ** : $\boldsymbol{p<0.01}$). Only the results of the colposcopy examination are used as prior knowledge in this table.

|  | ***Cervical-G1*** |  |  |  |
| --- | --- | --- | --- | --- |
|  | **Methods** | **AUC** (95% CI) | **Specificity** | **Sensitivity** |
| DT | ML | 0.637$\pm$0.037 [0.630 0.644] | 0.505$\pm$0.119 [0.481 0.528] | 0.705$\pm$0.132 [0.679 0.732] |
|  | PIML | 0.794$\pm$0.039 [0.787 0.802]($\uparrow$25%) ** | 0.804$\pm$0.245 [0.756 0.853] ** | 0.635$\pm$0.181 [0.599 0.671] ** |
|  | ML+ prior | 0.793$\pm$0.046 [0.783 0.802]($\uparrow$ 24%) ** | 0.844$\pm$0.246 [0.796 0.893] ** | 0.602$\pm$0.149 [0.572 0.631] ** |
| RF | ML | 0.679$\pm$0.036 [0.672 0.686] | 0.541$\pm$0.121 [0.517 0.565] | 0.704$\pm$0.132 [0.678 0.730] |
|  | PIML | 0.803$\pm$0.039 [0.795 0.811]($\uparrow$ 18%) ** | 0.800$\pm$0.234 [0.754 0.847] ** | 0.652$\pm$0.187 [0.615 0.689] * |
|  | ML+ prior | 0.821$\pm$0.040 [0.813 0.829]($\uparrow$ 21%) ** | 0.885$\pm$0.095 [0.866 0.904] ** | 0.593$\pm$0.107 [0.572 0.615] ** |
| SVM | ML | 0.638$\pm$0.037 [0.630 0.645] | 0.657$\pm$0.113 [0.635 0.679] | 0.560$\pm$0.126 [0.535 0.585] |
|  | PIML | 0.786$\pm$0.049 [0.777 0.796]($\uparrow$ 23%) ** | 0.820$\pm$0.211 [0.779 0.862] ** | 0.589$\pm$0.108 [0.548 0.630] |
|  | ML+ prior | 0.775$\pm$0.042 [0.767 0.783]($\uparrow$ 21%) ** | 0.926$\pm$0.078 [0.910 0.941] ** | 0.547$\pm$0.089 [0.529 0.564] ** |
| MLP | ML | 0.640$\pm$0.040 [0.632 0.648] | 0.538$\pm$0.133 [0.512 0.565] | 0.666$\pm$0.143 [0.638 0.695] |
|  | PIML | 0.797$\pm$0.039 [0.790 0.805]($\uparrow$ 25%) ** | 0.804$\pm$0.226 [0.759 0.848] ** | 0.648$\pm$0.174 [0.613 0.682] |
|  | ML+ prior | 0.797$\pm$0.042 [0.789 0.806]($\uparrow$ 25%) ** | 0.887$\pm$0.091 [0.869 0.905] ** | 0.569$\pm$0.097 [0.550 0.588] ** |
|  |  |  |  |  |
|  | ***Cervical-G2*** |  |  |  |
|  | **Methods** | **AUC** (95% CI) | **Specificity** | **Sensitivity** |
| DT | ML | 0.563$\pm$0.051 [0.553 0.573] | 0.639$\pm$0.122 [0.614 0.663] | 0.452$\pm$0.166 [0.419 0.485] |
|  | PIML | 0.741$\pm$0.027 [0.735 0.746]($\uparrow$ 32%) ** | 0.815$\pm$0.170 [0.781 0.849] ** | 0.569$\pm$0.140 [0.541 0.597] ** |
|  | ML+ prior | 0.746$\pm$0.028 [0.741 0.752]($\uparrow$ 33%) ** | 0.835$\pm$0.217 [0.792 0.878] ** | 0.551$\pm$0.132 [0.524 0.577] ** |
| RF | ML | 0.600$\pm$0.013 [0.597 0.602] | 0.720$\pm$0.101 [0.700 0.740] | 0.364$\pm$0.180 [0.328 0.399] |
|  | PIML | 0.744$\pm$0.030 [0.738 0.750]($\uparrow$ 24%) ** | 0.830$\pm$0.138 [0.803 0.857] ** | 0.567$\pm$0.134 [0.541 0.594] ** |
|  | ML+ prior | 0.779$\pm$0.010 [0.777 0.781]($\uparrow$ 30%) ** | 0.898$\pm$0.051 [0.888 0.908] ** | 0.502$\pm$0.070 [0.488 0.515] ** |
| SVM | ML | 0.583$\pm$0.026 [0.577 0.588] | 0.757$\pm$0.114 [0.735 0.780] | 0.345$\pm$0.146 [0.316 0.374] |
|  | PIML | 0.758$\pm$0.031 [0.752 0.764]($\uparrow$ 30%) ** | 0.815$\pm$0.173 [0.781 0.850] ** | 0.566$\pm$0.164 [0.533 0.598] ** |
|  | ML+ prior | 0.790$\pm$0.009 [0.788 0.792]($\uparrow$ 36%) ** | 0.833$\pm$0.107 [0.812 0.855] ** | 0.549$\pm$0.127 [0.524 0.575] ** |
| MLP | ML | 0.603$\pm$0.034 [0.596 0.610] | 0.695$\pm$0.113 [0.673 0.718] | 0.415$\pm$0.156 [0.384 0.446] |
|  | PIML | 0.760$\pm$0.029 [0.754 0.766]($\uparrow$ 26%) ** | 0.823$\pm$0.144 [0.795 0.852] ** | 0.587$\pm$0.139 [0.560 0.615] ** |
|  | ML+ prior | 0.781$\pm$0.029 [0.776 0.787]($\uparrow$ 30%) ** | 0.876$\pm$0.060 [0.864 0.888] ** | 0.557$\pm$0.093 [0.539 0.576] ** |

**Table S2 Performance comparison on Cervical-G1/G2 dataset (prior knowledge used: smear test).** All results are reported as mean value across 100 repetitions. ‘ML+prior’ refers to direct feature-level prior integration in ML. ‘PIML’ refers to our proposed Bayesian integration framework with ML. Asterisks indicate statistically significant improvement over baseline ML (Wilcoxon signed-rank test, * :$p<0.05$; ** : $p<0.01$). Only the results of the smear test are used as prior knowledge in this table.

|  | ***Cervical-G1*** |  |  |  |
| --- | --- | --- | --- | --- |
|  | **Methods** | **AUC** (95% CI) | **Specificity** | **Sensitivity** |
| DT | ML | 0.637$\pm$0.037 [0.630 0.644] | 0.505$\pm$0.119 [0.481 0.528] | 0.705$\pm$0.132 [0.679 0.732] |
|  | PIML | 0.833$\pm$0.032 [0.826 0.839]($\uparrow$ 31%) ** | 0.941$\pm$0.030 [0.935 0.947] ** | 0.660$\pm$0.059 [0.648 0.672] ** |
|  | ML+ prior | 0.810$\pm$0.036 [0.803 0.817]($\uparrow$ 27%) ** | 0.938$\pm$0.026 [0.933 0.943] ** | 0.661$\pm$0.060 [0.649 0.673] ** |
| RF | ML | 0.679$\pm$0.036 [0.672 0.686] | 0.541$\pm$0.121 [0.517 0.565] | 0.704$\pm$0.132 [0.678 0.730] |
|  | PIML | 0.838$\pm$0.033 [0.831 0.844]($\uparrow$ 23%) ** | 0.940$\pm$0.038 [0.933 0.948] ** | 0.661$\pm$0.063 [0.648 0.673] ** |
|  | ML+ prior | 0.852$\pm$0.032 [0.846 0.859]($\uparrow$ 25%) ** | 0.911$\pm$0.053 [0.901 0.922] ** | 0.668$\pm$0.069 [0.654 0.681] ** |
| SVM | ML | 0.638$\pm$0.037 [0.630 0.645] | 0.657$\pm$0.113 [0.635 0.679] | 0.560$\pm$0.126 [0.535 0.585] |
|  | PIML | 0.826$\pm$0.040 [0.818 0.834]($\uparrow$ 29%) ** | 0.906$\pm$0.148 [0.877 0.936] ** | 0.623$\pm$0.155 [0.592 0.654] ** |
|  | ML+ prior | 0.822$\pm$0.033 [0.816 0.829]($\uparrow$ 29%) ** | 0.917$\pm$0.041 [0.909 0.925] ** | 0.654$\pm$0.066 [0.641 0.667] ** |
| MLP | ML | 0.640$\pm$0.040 [0.632 0.648] | 0.538$\pm$0.133 [0.512 0.565] | 0.666$\pm$0.143 [0.638 0.695] |
|  | PIML | 0.835$\pm$0.032 [0.828 0.841]($\uparrow$ 30%) ** | 0.938$\pm$0.042 [0.930 0.946] ** | 0.662$\pm$0.062 [0.650 0.674] |
|  | ML+ prior | 0.828$\pm$0.033 [0.822 0.835]($\uparrow$ 29%) ** | 0.884$\pm$0.075 [0.869 0.899] ** | 0.650$\pm$0.079 [0.635 0.666] ** |
|  |  |  |  |  |
|  | ***Cervical-G2*** |  |  |  |
|  | **Methods** | **AUC** (95% CI) | **Specificity** | **Sensitivity** |
| DT | ML | 0.563$\pm$0.051 [0.553 0.573] | 0.639$\pm$0.122 [0.614 0.663] | 0.452$\pm$0.166 [0.419 0.485] |
|  | PIML | 0.781$\pm$0.024 [0.776 0.786]($\uparrow$ 39%) ** | 0.946$\pm$0.006 [0.944 0.947] ** | 0.527$\pm$0.005 [0.526 0.528] ** |
|  | ML+ prior | 0.750$\pm$0.021 [0.746 0.754]($\uparrow$ 33%) ** | 0.944$\pm$0.010 [0.942 0.946] ** | 0.527$\pm$0.011 [0.525 0.529] ** |
| RF | ML | 0.600$\pm$0.013 [0.597 0.602] | 0.720$\pm$0.101 [0.700 0.740] | 0.364$\pm$0.180 [0.328 0.399] |
|  | PIML | 0.793$\pm$0.036 [0.786 0.801]($\uparrow$ 32%) ** | 0.944$\pm$0.023 [0.939 0.948] ** | 0.529$\pm$0.026 [0.524 0.534] ** |
|  | ML+ prior | 0.816$\pm$0.010 [0.814 0.818]($\uparrow$ 36%) ** | 0.938$\pm$0.023 [0.934 0.943] ** | 0.505$\pm$0.061 [0.493 0.517] ** |
| SVM | ML | 0.583$\pm$0.026 [0.577 0.588] | 0.757$\pm$0.114 [0.735 0.780] | 0.345$\pm$0.146 [0.316 0.374] |
|  | PIML | 0.787$\pm$0.024 [0.782 0.791]($\uparrow$ 35%) ** | 0.917$\pm$0.139 [0.889 0.944] ** | 0.523$\pm$0.095 [0.504 0.542] ** |
|  | ML+ prior | 0.782$\pm$0.008 [0.781 0.784]($\uparrow$ 34%) ** | 0.889$\pm$0.070 [0.875 0.903] ** | 0.525$\pm$0.082 [0.509 0.542] ** |
| MLP | ML | 0.603$\pm$0.034 [0.596 0.610] | 0.695$\pm$0.113 [0.673 0.718] | 0.415$\pm$0.156 [0.384 0.446] |
|  | PIML | 0.790$\pm$0.027 [0.785 0.796]($\uparrow$ 31%) ** | 0.942$\pm$0.028 [0.937 0.948] ** | 0.529$\pm$0.023 [0.525 0.534] ** |
|  | ML+ prior | 0.798$\pm$0.025 [0.793 0.803]($\uparrow$ 32%) ** | 0.901$\pm$0.058 [0.890 0.913] ** | 0.527$\pm$0.083 [0.510 0.543] ** |
|  |  |  |  |  |

**Table S3 Optimal hyperparameter configurations for each model across three tasks.** This table summarizes the best-performing hyperparameter settings for each machine learning model—decision tree (DT), random forest (RF), support vector machine (SVM), and multilayer perceptron (MLP)—identified via grid search on the validation set for the three tasks: cervical neoplasia detection (Task 1), high-risk oral lesion detection (Task 2), and preterm birth detection (Task 3).

| Model | Hyper-para | Task 1 | Task 2 | Task 3 |
| --- | --- | --- | --- | --- |
| DT | MaxNumSplits | 30 | 5 | 15 |
|  | MinParentSize | 5 | 15 | 5 |
|  | MinLeafSize | 25 | 5 | 25 |
| RF | NumTrees | 80 | 60 | 20 |
|  | MinLeafSize | 20 | 5 | 5 |
|  | MaxNumSplits | 15 | 15 | 25 |
| SVM | KernelFunction | ‘rbf’ | ‘rbf’ | ‘rbf’ |
|  | BoxConstraint | 0.1 | 0.01 | 0.001 |
| MLP | Layer Setting | [10] | [10 10] | [20] |

**Table S4.1 Exact P-values for selected pairwise comparisons discussed in the main text about task 1.**

| **Task** | **Setting** | **Metric** | **Comparison** | **P-Value** | **Significance** |
| --- | --- | --- | --- | --- | --- |
| Cervical-G1 | DT | AUC | PIML vs Prior | 4.1e-18 | ** |
|  |  | Specificity |  | 0.135 | NS |
|  |  | Sensitivity |  | 0.125 | NS |
|  | RF | AUC |  | 4.0e-18 | ** |
|  |  | Specificity |  | 0.126 | NS |
|  |  | Sensitivity |  | 0.180 | NS |
|  | SVM | AUC |  | 5.0e-18 | ** |
|  |  | Specificity |  | 0.208 | NS |
|  |  | Sensitivity |  | 0.004 | * |
|  | MLP | AUC |  | 3.9e-18 | ** |
|  |  | Specificity |  | 0.125 | NS |
|  |  | Sensitivity |  | 0.161 | NS |
|  | DT | AUC | PIML vs ML | 3.9e-18 | ** |
|  |  | Specificity |  | 4.0e-18 | ** |
|  |  | Sensitivity |  | 7.5e-04 | ** |
|  | RF | AUC |  | 3.8e-18 | ** |
|  |  | Specificity |  | 4.0e-18 | ** |
|  |  | Sensitivity |  | 0.008 | ** |
|  | SVM | AUC |  | 3.9e-18 | ** |
|  |  | Specificity |  | 3.5e-15 | ** |
|  |  | Sensitivity |  | 1.0e-04 | ** |
|  | MLP | AUC |  | 3.9e-18 | ** |
|  |  | Specificity |  | 4.2e-18 | ** |
|  |  | Sensitivity |  | 0.709 | NS |
| Cervical-G2 | DT | AUC | PIML vs Prior | 3.9e-18 | ** |
|  |  | Specificity |  | 2.5e-23 | ** |
|  |  | Sensitivity |  | 2.5e-23 | ** |
|  | RF | AUC |  | 3.8e-18 | ** |
|  |  | Specificity |  | 2.2e-22 | ** |
|  |  | Sensitivity |  | 1.6e-07 | ** |
|  | SVM | AUC |  | 3.9e-18 | ** |
|  |  | Specificity |  | 3.3e-20 | ** |
|  |  | Sensitivity |  | 2.0e-20 | ** |
|  | MLP | AUC |  | 3.8e-18 | ** |
|  |  | Specificity |  | 3.9e-18 | ** |
|  |  | Sensitivity |  | 3.9e-23 | ** |
|  | DT | AUC | PIML vs ML | 3.9e-18 | ** |
|  |  | Specificity |  | 4.3e-18 | ** |
|  |  | Sensitivity |  | 1.9e-04 | ** |
|  | RF | AUC |  | 3.9e-18 | ** |
|  |  | Specificity |  | 4.0e-18 | ** |
|  |  | Sensitivity |  | 1.7e-11 | ** |
|  | SVM | AUC |  | 3.8e-18 | ** |
|  |  | Specificity |  | 8.2e-15 | ** |
|  |  | Sensitivity |  | 1.2e-13 | ** |
|  | MLP | AUC |  | 3.8e-18 | ** |
|  |  | Specificity |  | 4.5e-18 | ** |
|  |  | Sensitivity |  | 9.5e-09 | ** |

**Table S4.2 Exact P-values for selected pairwise comparisons discussed in the main text about task 1.**

| **Task** | **Setting** | **Metric** | **Comparison** | **P-Value** | **Significance** |
| --- | --- | --- | --- | --- | --- |
| Cervical-G1 | DT | AUC | PIML vs ML+prior | 3.9e-18 | ** |
|  |  | Specificity |  | 3.9e-18 | ** |
|  |  | Sensitivity |  | 8.9e-04 | ** |
|  | RF | AUC |  | 3.9e-18 | ** |
|  |  | Specificity |  | 3.9e-18 | ** |
|  |  | Sensitivity |  | 0.008 | ** |
|  | SVM | AUC |  | 3.9e-18 | ** |
|  |  | Specificity |  | 4.1e-18 | ** |
|  |  | Sensitivity |  | 1.9e-09 | ** |
|  | MLP | AUC |  | 3.9e-18 | ** |
|  |  | Specificity |  | 3.8e-18 | ** |
|  |  | Sensitivity |  | 0.226 | NS |
| Cervical-G2 | DT | AUC | PIML vs ML+prior | 3.9e-18 | ** |
|  |  | Specificity |  | 4.1e-18 | ** |
|  |  | Sensitivity |  | 1.8e-04 | ** |
|  | RF | AUC |  | 3.9e-18 | ** |
|  |  | Specificity |  | 3.8e-18 | ** |
|  |  | Sensitivity |  | 1.8e-09 | ** |
|  | SVM | AUC |  | 3.9e-18 | ** |
|  |  | Specificity |  | 4.4e-14 | ** |
|  |  | Sensitivity |  | 4.0e-14 | ** |
|  | MLP | AUC |  | 3.9e-18 | ** |
|  |  | Specificity |  | 1.3e-17 | ** |
|  |  | Sensitivity |  | 1.1e-07 | ** |

**Table S5 Exact P-values for selected pairwise comparisons discussed in the main text about task 2.**

| **Task** | **Setting** | **Metric** | **Comparison** | **P-Value** | **Significance** |
| --- | --- | --- | --- | --- | --- |
| Oral Cancer | DT | AUC | PIML vs ML | 7.6e-05 | ** |
|  |  | Specificity |  | 3.4e-04 | ** |
|  |  | Sensitivity |  | 0.003 | ** |
|  | RF | AUC |  | 0.032 | * |
|  |  | Specificity |  | 1.7e-04 | ** |
|  |  | Sensitivity |  | 0.444 | NS |
|  | SVM | AUC |  | 0.643 | NS |
|  |  | Specificity |  | 1.4e-05 | ** |
|  |  | Sensitivity |  | 0.674 | NS |
|  | MLP | AUC |  | 0.804 | NS |
|  |  | Specificity |  | 1.2e-04 | ** |
|  |  | Sensitivity |  | 0.886 | NS |
|  | DT | AUC | PIML vs Prior | 0.008 | ** |
|  |  | Specificity |  | 2.2e-05 | ** |
|  |  | Sensitivity |  | 3.2e-07 | ** |
|  | RF | AUC |  | 0.025 | * |
|  |  | Specificity |  | 0.015 | * |
|  |  | Sensitivity |  | 2.2e-07 | ** |
|  | SVM | AUC |  | 3.7e-05 | ** |
|  |  | Specificity |  | 0.087 | NS |
|  |  | Sensitivity |  | 7.4e-08 | ** |
|  | MLP | AUC |  | 3.9e-05 | ** |
|  |  | Specificity |  | 0.039 | * |
|  |  | Sensitivity |  | 9.9e-10 | ** |
|  | DT | AUC | PIML vs ML+prior | 2.4e-05 | ** |
|  |  | Specificity |  | 1.2e-04 | ** |
|  |  | Sensitivity |  | 0.008 | ** |
|  | RF | AUC |  | 0.006 | ** |
|  |  | Specificity |  | 0.004 | ** |
|  |  | Sensitivity |  | 0.590 | NS |
|  | SVM | AUC |  | 1.9e-11 | ** |
|  |  | Specificity |  | 0.556 | NS |
|  |  | Sensitivity |  | 0.091 | NS |
|  | MLP | AUC |  | 0.627 | NS |
|  |  | Specificity |  | 0.010 | * |
|  |  | Sensitivity |  | 0.755 | NS |

**Table S6 Exact P-values for selected pairwise comparisons discussed in the main text about task 3.**

| **Task** | **Setting** | **Metric** | **Comparison** | **P-Value** | **Significance** |
| --- | --- | --- | --- | --- | --- |
| Preterm birth diagnosis | DT | AUC | PIML vs ML | 8.2e-18 | ** |
|  |  | Specificity |  | 1.5e-16 | ** |
|  |  | Sensitivity |  | 1.4e-14 | ** |
|  | RF | AUC |  | 2.0e-17 | ** |
|  |  | Specificity |  | 2.6e-12 | ** |
|  |  | Sensitivity |  | 0.003 | ** |
|  | SVM | AUC |  | 2.8e-16 | ** |
|  |  | Specificity |  | 0.500 | NS |
|  |  | Sensitivity |  | 0.012 | * |
|  | MLP | AUC |  | 2.2e-16 | ** |
|  |  | Specificity |  | 5.8e-12 | ** |
|  |  | Sensitivity |  | 0.255 | NS |
|  | DT | AUC | PIML vs Prior | 0.144 | NS |
|  |  | Specificity |  | 0.673 | NS |
|  |  | Sensitivity |  | 0.900 | NS |
|  | RF | AUC |  | 0.023 | * |
|  |  | Specificity |  | 0.021 | * |
|  |  | Sensitivity |  | 4.6e-05 | ** |
|  | SVM | AUC |  | 2.6e-14 | ** |
|  |  | Specificity |  | 0.423 | NS |
|  |  | Sensitivity |  | 2.4e-09 | ** |
|  | MLP | AUC |  | 1.1e-04 | ** |
|  |  | Specificity |  | 9.7e-04 | ** |
|  |  | Sensitivity |  | 7.1e-07 | ** |
|  | DT | AUC | PIML vs ML+prior | 2.5e-13 | ** |
|  |  | Specificity |  | 3.2e-07 | ** |
|  |  | Sensitivity |  | 0.046 | * |
|  | RF | AUC |  | 4.5e-04 | ** |
|  |  | Specificity |  | 0.002 | ** |
|  |  | Sensitivity |  | 0.464 | NS |
|  | SVM | AUC |  | 0.003 | ** |
|  |  | Specificity |  | 0.160 | NS |
|  |  | Sensitivity |  | 3.2e-05 | ** |
|  | MLP | AUC |  | 0.105 | NS |
|  |  | Specificity |  | 0.024 | * |
|  |  | Sensitivity |  | 0.016 | * |

**Table S7 Additional sensitivity analysis of the oral high-risk lesion dataset using 10 repeated stratified nested patient-level 3-fold cross-validation.**

|  | **Methods** | **AUC** (95% CI) | **Specificity** | **Sensitivity** |
| --- | --- | --- | --- | --- |
|  | **Prior** | 0.774$\pm$0.022 [0.740 0.797] | 0.887$\pm$0.001 [0.867 0.894] | 0.778$\pm$0.031 [0.766 0.797] |
| DT | ML | 0.763$\pm$0.060 [0.673 0.826] | 0.677$\pm$0.323 [0.452 0.813] | 0.656$\pm$0.192 [0.433 0.876] |
|  | PIML | 0.796$\pm$0.056 [0.690 0.856] | 0.925$\pm$0.025 [0.887 0.962] | 0.644$\pm$0.102 [0.444 0.778] |
|  | ML+ prior | 0.765$\pm$0.060 [0.673 0.826] | 0.738$\pm$0.255 [0.421 0.881] | 0.622$\pm$0.130 [0.481 0.762] |
| MLP | ML | 0.808$\pm$0.079 [0.629 0.892] | 0.847$\pm$0.071 [0.660 0.906] | 0.622$\pm$0.119 [0.333 0.778] |
|  | PIML | 0.818$\pm$0.048 [0.751 0.885] | 0.891$\pm$0.045 [0.830 0.962] | 0.700$\pm$0.092 [0.556 0.778] |
|  | ML+ prior | 0.756$\pm$0.046 [0.675 0.822] | 0.876$\pm$0.095 [0.698 0.981] | 0.556$\pm$0.181 [0.376 0.709] |

To assess whether the findings from the primary oral-lesion experiment were dependent on the repeated 8:1:1 hold-out strategy, an additional sensitivity analysis was performed using 10 repeated stratified nested patient-level 3-fold cross-validation. DT and MLP were selected as representative classifiers. All data splitting was performed at the patient level, and repeated EIS readings from the same patient were retained within the same fold. For evaluation, prediction scores from repeated readings were averaged to generate a single patient-level score.

The results showed an overall pattern consistent with the primary analysis. For DT, PIML achieved an AUC of 0.796 ± 0.056, compared with 0.763 ± 0.060 for baseline ML, while specificity increased markedly from 0.677 ± 0.323 to 0.925 ± 0.025. ML+prior produced an AUC of 0.765 ± 0.060 and specificity of 0.738 ± 0.255. Although DT sensitivity showed some variability, the principal improvement in AUC and specificity observed with PIML was retained under the alternative validation strategy.

For MLP, PIML achieved an AUC of 0.818 ± 0.048, compared with 0.808 ± 0.079 for baseline ML and 0.756 ± 0.046 for ML+prior. Specificity increased from 0.847 ± 0.071 for ML to 0.891 ± 0.045 for PIML, while sensitivity increased from 0.622 ± 0.119 to 0.700 ± 0.092. These results were consistent with the main experiment, in which PIML also provided improved diagnostic performance over the corresponding baseline ML model.

Overall, the additional nested patient-level cross-validation analysis supports the robustness of the main findings and indicates that the observed benefit of PIML is not specific to the original repeated hold-out partitioning strategy.

**Table S8. External calibration analysis of PIML Bayesian-updated probability estimates on the Cervical-G2 cohort across 100 repeated experiments.**

| Group | ML Evidence | Smear | Colposcopy | Mean PIML Probability | Mean Observed Disease Rate |
| --- | --- | --- | --- | --- | --- |
| 1 | 0 | 0 | 0 | 0.071$\pm$0.019 | 0.061$\pm$0.018 |
| 2 | 0 | 0 | 1 | 0.566$\pm$0.295 | 0.323$\pm$0.216 |
| 3 | 0 | 1 | 0 | 0.420$\pm$0.077 | 0.495$\pm$0.172 |
| 4 | 0 | 1 | 1 | 0.894$\pm$0.043 | 0.776$\pm$0.042 |
| 5 | 1 | 0 | 0 | 0.208$\pm$0.030 | 0.135$\pm$0.056 |
| 6 | 1 | 0 | 1 | 0.744$\pm$0.206 | 0.432$\pm$0.142 |
| 7 | 1 | 1 | 0 | 0.708$\pm$0.034 | 0.771$\pm$0.115 |
| 8 | 1 | 1 | 1 | 0.968$\pm$0.007 | 0.888$\pm$0.111 |

**Table S9. Mean absolute calibration error for the eight PIML groups in the Cervical-G2 cohort.**

| Group | ML Evidence | Smear | Colposcopy | Mean Error |
| --- | --- | --- | --- | --- |
| 1 | 0 | 0 | 0 | 0.02$\pm$0.01 |
| 2 | 0 | 0 | 1 | 0.28$\pm$0.29 |
| 3 | 0 | 1 | 0 | 0.22$\pm$0.19 |
| 4 | 0 | 1 | 1 | 0.12$\pm$0.05 |
| 5 | 1 | 0 | 0 | 0.08$\pm$0.05 |
| 6 | 1 | 0 | 1 | 0.43$\pm$0.22 |
| 7 | 1 | 1 | 0 | 0.17$\pm$0.28 |
| 8 | 1 | 1 | 1 | 0.10$\pm$0.08 |

Patients in the Cervical-G2 external cohort were categorized according to the binary ML diagnostic evidence, smear-test result, and colposcopy result, resulting in eight possible combinations. For each category, the PIML Bayesian-updated probability estimate was compared with the corresponding observed disease rate in the external cohort. Results summarize the calibration analysis across 100 repeated experiments. ML evidence refers to the binary diagnostic output of the baseline ML classifier, where ML = 1 indicates a positive prediction for cervical neoplasia and ML = 0 indicates a negative prediction. A decision tree (DT) model was used as the representative ML classifier for this calibration analysis. The reported PIML probability represents the Bayesian-updated estimated probability of cervical neoplasia for patients belonging to each corresponding combination of ML evidence, smear-test result, and colposcopy result.

**Table S8** presents the external calibration analysis of the PIML Bayesian-updated probability estimates on the Cervical-G2 cohort across 100 repeated experiments. Patients were stratified into eight groups according to the binary ML diagnostic evidence, smear-test result, and colposcopy result. For each group, the mean PIML-estimated probability of cervical neoplasia was compared with the corresponding mean observed disease rate. Several groups showed relatively close agreement between the estimated and observed probabilities. For example, the estimated and observed probabilities were 0.071 versus 0.061 for Group 1, 0.420 versus 0.495 for Group 3, 0.708 versus 0.771 for Group 7, and 0.968 versus 0.888 for Group 8. Larger discrepancies were observed for some combinations, particularly Groups 2 and 6, indicating that the degree of probability calibration varied across different combinations of ML evidence and clinical prior information.

**Table S9** further quantifies the discrepancy between the PIML probability estimate and the observed disease rate using the absolute calibration error, calculated as the absolute difference between the two values within each repetition and then summarized across the 100 repeated experiments. The mean absolute error varied across the eight groups, ranging from 0.02 ± 0.01 in Group 1 to 0.43 ± 0.22 in Group 6. Relatively small errors were also observed in Groups 4, 5, and 8, whereas Groups 2, 3, 6, and 7 showed greater variability or larger discrepancies. These results suggest that the Bayesian-updated probability estimates show good agreement with observed disease frequencies in several clinical-evidence combinations, while calibration remains less consistent in some subgroups. The relatively unstable results observed in Groups 2 and 6 can be partly explained by the small number (5 in Group 2 and 8 in Group 6) of patients satisfying these specific combinations of ML evidence, smear-test result, and colposcopy result, leading to unavoidable inaccuracies in frequency-based probability estimates.

# 2 Supplementary appendix

**Appendix 1**

In the Cervical-G1 dataset which used as the primary training and internal evaluation cohort, PIML consistently outperformed the baseline ML models across all major performance metrics. In addition, when compared with the prior-only method, PIML demonstrated substantial improvements in both discriminative ability and diagnostic balance. While the prior-only approach achieved an AUC of 0.751 [95% CI: 0.743–0.758] with limited specificity (0.631) and sensitivity (0.681), PIML significantly improved AUC across all models (p<0.01, Supplementary Table 4.1) while achieving a more balanced trade-off between sensitivity and specificity.

For instance, when compared with the ML only, with the decision tree (DT) classifier, PIML improved AUC from 0.637 [95% CI: 0.630-0.644] to 0.842 [95% CI: 0.835 0.848] (reflecting a 32% gain, p<0.01), and improved specificity from 0.505 [95% CI: 0.481-0.528] to 0.946 [95% CI: 0.942-0.950]. Compared with the prior-only method, this corresponds to a substantial improvement in AUC from 0.750 to 0.842 (p<0.01), together with a marked increase in sensitivity (from 0.514 to 0.650), indicating that prior knowledge alone is insufficient for reliable diagnosis. Similar trends were observed across all other classifiers: RF showed a 26% AUC increase (0.679 to 0.846, p<0.01), SVM showed a 28% AUC improvement (0.638 to 0.817, p<0.01), MLP showed a 31% AUC gain (0.640 to 0.843, p<0.01), with significant gains in both specificity and sensitivity (p<0.01 in most cases, Supplementary Table 4.1).

When compared with the ML+prior approach, PIML further demonstrated consistent advantages across all classifiers, with statistically significant improvements (p<0.01, Supplementary Table 4.2). For example, in the DT model, PIML achieved a higher AUC than ML+prior (0.842 vs 0.819, p<0.01), along with improved specificity (0.946 vs 0.934). Similarly, in the MLP model, PIML improved AUC from 0.833 to 0.843 (p<0.01), and substantially increased specificity from 0.894 to 0.944. These results indicate that Bayesian integration enables more effective utilization of prior knowledge compared to direct feature concatenation.

Results from the external cohort, Cervical-G2, further confirmed the generalizability of the PIML framework. Despite the smaller and limited sample size (n=112), all classifiers demonstrated substantial improvements in both AUC and classification performance when PIML was applied. For instance, AUC for SVM increased from 0.583 [95% CI: 0.577-0.588] to 0.804 [95% CI: 0.799-0.809] (gain: 38%, p<0.01), and sensitivity improved from 0.345 [95% CI: 0.316-0.374] to 0.843 [95% CI: 0.827-0.859]. These gains were consistently statistically significant (p<0.01, Supplementary Table 4.2), underscoring the robustness of PIML and generalizes well across cohorts. Notably, similar improvements were also observed when compared with the prior-only method (p<0.01, Supplementary Table 4.2), indicating that the advantage of PIML is not limited to data-driven learning but extends to scenarios where prior knowledge alone is insufficient.

To further examine the individual contribution of each type of prior knowledge, we performed ablation experiments in which either colposcopic assessment or smear test results were exclusively incorporated during model training and Bayesian inference. The corresponding results are provided in Supplementary Tables 1, 2 and Fig.3. Notably, both single-prior configurations also yielded substantial improvements over the baseline ML models across all metrics and classifiers. For example, in the Cervical-G1 dataset using the DT model, inclusion of only smear test results improved the AUC from 0.637 (95% CI: [0.630, 0.644]) to 0.833 (95% CI: [0.826, 0.839]), whereas using only colposcopic assessments yielded an AUC of 0.794 (95% CI: [0.787, 0.802]). A comparable trend was observed in the Cervical-G2 cohort, where inclusion of only smear test priors in the PIML model yielded an AUC of 0.781 (95% CI: [0.776, 0.786]) in DT, compared to 0.741 (95% CI: [0.735, 0.746]) when only colposcopic information was used. These results underscore the individual utility of each prior knowledge source, while collectively supporting the synergistic benefit of their combined use—as evidenced in the main experiments—through complementary knowledge integration.

**Appendix 2**

As summarized in Table 3, patient-level evaluation demonstrated that the effect of prior-information integration varied across the different ML classifiers. For the DT model, PIML achieved an AUC of 0.822 [95% CI: 0.789–0.855], representing an approximately 13% relative improvement over the baseline ML model (AUC = 0.726 [0.687–0.766]). The improvement was particularly evident in specificity, which increased substantially from 0.748 [0.664–0.833] for ML to 0.930 [0.908–0.952] for PIML. Sensitivity also increased from 0.535 [0.457–0.613] to 0.635 [0.566–0.704]. In comparison, direct feature-level integration of smoking status through ML+prior resulted in an AUC of 0.721 [0.681–0.762], a specificity of 0.733 [0.648–0.819], and a sensitivity of 0.545 [0.468–0.622]. These results indicate that, for DT, Bayesian integration of the patient-specific prior provided a clearer advantage than directly including smoking status as an additional model feature.

A similar pattern was observed for RF. PIML increased the AUC from 0.785 [0.746–0.824] for baseline ML to 0.848 [0.812–0.883], corresponding to an approximately 8% relative improvement. Sensitivity also increased from 0.555 [0.474–0.636] to 0.607 [0.548–0.650]. The specificity of PIML increased from 0.783 [0.720–0.847] to 0.842 [0.793–0.891]. However, ML+prior achieved the highest specificity for RF, reaching 0.912 [0.875–0.948], while its AUC was 0.803 [0.762–0.845]. Therefore, although both approaches benefited from the incorporation of smoking information, PIML provided the highest overall discrimination in terms of AUC, whereas direct feature-level integration produced the highest specificity for RF.

For SVM, a different pattern was observed. Baseline ML achieved an AUC of 0.756 [0.712–0.800], whereas PIML produced an AUC of 0.741 [0.701–0.780]. Nevertheless, PIML substantially increased specificity from 0.718 [0.648–0.788] to 0.900 [0.866–0.933], while maintaining a similar sensitivity (0.547 [0.472–0.621] versus 0.540 [0.458–0.622] for baseline ML). In contrast, ML+prior achieved the strongest overall performance for SVM, with an AUC of 0.909 [0.886–0.932], representing an approximately 20% improvement over baseline ML, together with a specificity of 0.882 [0.843–0.921] and a sensitivity of 0.625 [0.550–0.700]. These findings suggest that SVM benefited more strongly from direct feature-level incorporation of smoking status, whereas the main advantage of PIML for this classifier was the substantial improvement in specificity.

For MLP, PIML achieved an AUC of 0.772 [0.725–0.817], compared with 0.752 [0.706–0.797] for baseline ML and 0.755 [0.709–0.802] for ML+prior. This corresponds to an approximately 3% relative improvement over the baseline ML model. More prominently, specificity increased from 0.790 [0.735–0.846] for ML to 0.912 [0.884–0.940] for PIML, while sensitivity increased from 0.510 [0.423–0.577] to 0.552 [0.431–0.634]. ML+prior also increased specificity to 0.842 [0.788–0.896], but the improvement was smaller than that observed with PIML.

The prior-only method achieved an AUC of 0.827, a specificity of 0.873, and a sensitivity of 0.700. This relatively strong performance reflects the clinically established relationship between smoking status and oral lesion risk. Importantly, however, the classifier-based experiments demonstrate that the way in which prior information is incorporated can lead to different diagnostic trade-offs. PIML achieved higher specificity than the prior-only approach for DT, SVM, and MLP, while RF-PIML achieved the highest AUC among the RF-based strategies.

Overall, the patient-level results demonstrate that the benefit of prior-information integration is classifier dependent. PIML improved AUC relative to baseline ML for DT, RF, and MLP and consistently increased specificity for all four classifiers. For DT, RF, and MLP, PIML also increased sensitivity relative to the corresponding ML baseline. SVM represented an exception in terms of overall discrimination, for which direct feature-level integration through ML+prior achieved the highest AUC and sensitivity. These findings indicate that PIML is particularly effective in improving diagnostic specificity while maintaining or improving sensitivity for several different ML backbones.

**Appendix 3**

Table 4 summarizes the patient-level performance of the different strategies on the PTB dataset. Overall, PIML substantially improved the AUC of the corresponding baseline ML models across all four classifiers, while also producing a more balanced trade-off between sensitivity and specificity.

For the DT model, baseline ML achieved an AUC of 0.498 [95% CI: 0.496–0.501]. PIML increased the AUC to 0.704 [0.685–0.723], corresponding to an approximately 41% improvement. ML+prior also improved AUC to 0.605 [0.583–0.627], whereas PIML(APP) achieved an AUC of 0.692 [0.675–0.711]. The difference was particularly pronounced in specificity. Baseline DT produced a specificity of only 0.005 [0.002–0.012] together with an extremely high sensitivity of 0.993 [0.983–1.000], indicating a strong tendency to classify subjects as positive. In contrast, PIML increased specificity to 0.610 [0.567–0.652] while maintaining a sensitivity of 0.754 [0.718–0.791]. PIML(APP) showed a similar balance, with specificity of 0.600 [0.562–0.631] and sensitivity of 0.745 [0.723–0.781].

For RF, baseline ML achieved an AUC of 0.448 [0.425–0.471]. PIML increased this to 0.693 [0.661–0.705], corresponding to an approximately 52% improvement. ML+prior also substantially improved AUC to 0.657 [0.636–0.678], while PIML(APP) achieved an AUC of 0.676 [0.652–0.699]. PIML increased specificity from 0.358 [0.308–0.407] to 0.649 [0.617–0.681] and sensitivity from 0.576 [0.521–0.630] to 0.679 [0.631–0.726]. PIML(APP) showed very similar specificity and sensitivity of 0.648 [0.614–0.683] and 0.664 [0.616–0.713], respectively.

For SVM, baseline ML achieved an AUC of 0.465 [0.442–0.488]. PIML increased the AUC to 0.666 [0.643–0.690], representing an approximately 43% improvement, while PIML(APP) achieved an AUC of 0.653 [0.629–0.678], corresponding to an approximately 40% improvement. PIML also increased sensitivity from 0.454 [0.387–0.521] to 0.558 [0.504–0.611]. In contrast, direct feature-level integration through ML+prior did not improve overall discrimination, with an AUC of 0.460 [0.431–0.488], although its specificity increased to 0.593 [0.530–0.656].

For MLP, baseline ML achieved an AUC of 0.488 [0.465–0.511]. PIML increased the AUC to 0.680 [0.649–0.691], corresponding to an approximately 37% improvement. ML+prior achieved an AUC of 0.651 [0.630–0.671], while PIML(APP) achieved 0.664 [0.643–0.685]. Specificity increased substantially from 0.394 [0.347–0.442] for baseline ML to 0.668 [0.635–0.700] for PIML and 0.670 [0.639–0.701] for PIML(APP). PIML also increased sensitivity from 0.594 [0.538–0.651] to 0.650 [0.603–0.697].

The prior-only method achieved an AUC of 0.685 [0.667–0.724], with specificity of 0.608 [0.566–0.651] and sensitivity of 0.757 [0.721–0.794]. These results indicate that previous PTB history alone provides substantial prognostic information. Therefore, the value of PIML should not be interpreted solely in terms of outperforming the prior-only approach in every classifier. Rather, its principal advantage lies in systematically integrating clinically meaningful prior information with EIS-derived ML evidence, thereby substantially improving classifier performance and the sensitivity–specificity balance, while providing more reliable PTB prediction than other ML-based approaches.

Importantly, the performance of PIML(APP) was consistently comparable to that of PIML incorporating externally estimated priors. For DT, RF, SVM, and MLP, the corresponding AUC values were 0.692, 0.676, 0.653, and 0.664 for PIML(APP), compared with 0.704, 0.693, 0.666, and 0.680, respectively, for PIML.This close agreement provides evidence that the proposed Bayesian framework can flexibly incorporate externally derived, patient-specific prior information rather than relying exclusively on prior probabilities estimated from the training cohort. This flexibility addresses a key limitation of conventional ML approaches, which typically depend solely on information derived from the training data.

Overall, the patient-level PTB results demonstrate that PIML consistently improved AUC relative to baseline ML across all four classifiers and generally produced a more balanced sensitivity-specificity profile. PIML(APP) yielded highly comparable results, further supporting the flexibility of the proposed framework for integrating prior knowledge from heterogeneous clinical sources.

# 3 Supplementary case

This example illustrates how PIML converts a patient-specific clinical prior and an ML-derived diagnostic result into a posterior probability of disease. The numerical values below are hypothetical and are provided solely to demonstrate the interpretation of the Bayesian updating process; they are not derived from an individual participant in the present datasets.

Consider a patient whose available clinical information yields a prior probability of disease of 0.30. Assume that the ML classifier has a sensitivity of 0.80 and a specificity of 0.90, estimated from the validation data according to the procedure described in Section 2.1.5 of the main manuscript.

If the ML classifier predicts the patient as positive, the posterior probability is calculated as:

$P\left( H | E \right)=\frac{0.8\times0.3}{0.8\times0.3+(1-0.9)\times0.7}=0.774$

The patient begins with an estimated disease probability of 30% based on available clinical information. After receiving a positive ML result from a classifier with 80% sensitivity and 90% specificity, the estimated probability of disease increases to approximately 77.4%. Thus, the ML output is interpreted as additional diagnostic evidence that substantially increases the patient-specific probability of disease.

If the same ML classifier instead predicts the patient as negative, the posterior probability is calculated as:

$P\left( H | \bar{E} \right)=\frac{0.2\times0.3}{0.2\times0.3+0.9\times0.7}=0.087$

Under the same initial clinical risk, a negative ML result decreases the estimated probability of disease from 30% to approximately 8.7%. The posterior probability therefore provides a transparent quantitative representation of how the ML evidence modifies the initial clinical assessment.

This example illustrates the concept of “clinical interpretability” within the PIML framework. Rather than seeking to explain the internal feature representations of the underlying ML classifier, PIML provides a transparent and traceable diagnostic pathway in which the patient-specific prior probability, ML-derived diagnostic evidence, and the validated sensitivity and specificity associated with that evidence are explicitly and coherently integrated to calculate the posterior probability of the disease of concern. Thus, the final diagnostic output is presented as an explicit probabilistic update, rather than as an isolated binary prediction from an ML classifier.
